# Compartmentalised expression of *Delta-like 1 *in epithelial somites is required for the formation of intervertebral joints

**DOI:** 10.1186/1471-213X-7-68

**Published:** 2007-06-17

**Authors:** Ingeborg Teppner, Sonja Becker, Martin Hrabé de Angelis, Achim Gossler, Johannes Beckers

**Affiliations:** 1 GSF – National Research Center for Environment and Health, GmbH, Institute of Experimental Genetics, Ingolstaedter Landstr.1, 85764 Neuherberg, Germany; 2 Medizinische Hochschule Hannover, Institute for Molecular Biology, Carl-Neuberg-Str.1, 30625 Hannover, Germany

## Abstract

**Background:**

Expression of the mouse *Delta-like 1 *(*Dll1*) gene in the presomitic mesoderm and in the caudal halves of somites of the developing embryo is required for the formation of epithelial somites and for the maintenance of caudal somite identity, respectively. The rostro-caudal polarity of somites is initiated early on within the presomitic mesoderm in nascent somites. Here we have investigated the requirement of restricted *Dll1 *expression in caudal somite compartments for the maintenance of rostro-caudal somite polarity and the morphogenesis of the axial skeleton. We did this by overexpressing a functional copy of the *Dll1 *gene throughout the paraxial mesoderm, in particular in anterior somite compartments, during somitogenesis in transgenic mice.

**Results:**

Epithelial somites were generated normally and appeared histologically normal in embryos of two independent *Dll1 *over-expressing transgenic lines. Gene expression analyses of rostro-caudal marker genes suggested that over-expression of *Dll1 *without restriction to caudal compartments was not sufficient to confer caudal identity to rostral somite halves in transgenic embryos. Nevertheless, *Dll1 *over-expression caused dysmorphologies of the axial skeleton, in particular, in morphological structures that derive from the articular joint forming compartment of vertebrae. Accordingly, transgenic animals exhibited missing or reduced intervertebral discs, rostral and caudal articular processes as well as costal heads of ribs. In addition, the midline of the vertebral column did not develop normally. Transgenic mice had open neural arches and split vertebral bodies with ectopic pseudo-growth plates. Endochondral bone formation and ossification in the developing vertebrae were delayed.

**Conclusion:**

The mice overexpressing *Dll1 *exhibit skeletal dysmorphologies that are also evident in several mutant mice with defects in somite compartmentalisation. The *Dll1 *transgenic mice demonstrate that vertebral dysmorphologies such as bony fusions of vertebrae and midline vertebral defects can occur without apparent changes in somitic rostro-caudal marker gene expression. Also, we demonstrate that the over-expression of the *Dll1 *gene in rostral epithelial somites is not sufficient to confer caudal identity to rostral compartments. Our data suggest that the restricted *Dll1 *expression in caudal epithelial somites may be particularly required for the proper development of the intervertebral joint forming compartment.

## Background

Segmentation along the rostro-caudal (R/C) axis is a fundamental characteristic of vertebrates. It originates during embryogenesis when the paraxial mesoderm is divided bilaterally into spheres of epithelial somites, which enclose a core of mesenchymal cells, the somitocoele cells [1]. Each somite is divided in a rostral and caudal compartment with distinct gene expression and developmental fate [2-4]. This R/C somite polarity is established early on in the presomitic mesoderm (PSM) prior to segmentation [5-7] and is essential for the subsequent resegmentation of sclerotomes [8,9] and the sequential patterning of the neural tube [10,11].

Experimental evidence suggests that *Mesp2 *and *Notch *signalling are required for the initiation of R/C somite compartmentalisation in nascent somites through induction and suppression of *Dll1 *expression in caudal and rostral somite halves, respectively [12-14]. The maintenance of somite R/C polarity requires the compartmentalised expression of caudal genes, such as *Dll1*, *Notch1*, *Paraxis *and *Uncx4.1 *[15].

Here, we analysed the requirement of restricted *Dll1 *expression in epithelial somites for the maintenance of R/C identity following the initial establishment and for the development of somitocoele cells, in particular. It has been demonstrated that somitocoele cells contribute to proximal ribs, the articular surface of intervertebral (zygapophyseal) joints, and the peripheral parts of the intervertebral discs (IVDs) [16,17]. In the avian embryo, these cells constitute a joint forming compartment, the arthrotome [18,19]. The molecular mechanisms underlying the specification of the arthrotome compartment have not been studied. Our data demonstrate that the over-expression of *Dll1 *throughout epithelial somites of transgenic mice does not alter somite polarity but affects the development of intervertebral joints, IVDs and proximal ribs. This suggests a role of restricted *Dll1 *expression in caudal epithelial somites for arthrotome development.

## Results

### Generation of *Dll1 *gain-of-function transgenic lines

**Figure 1:**
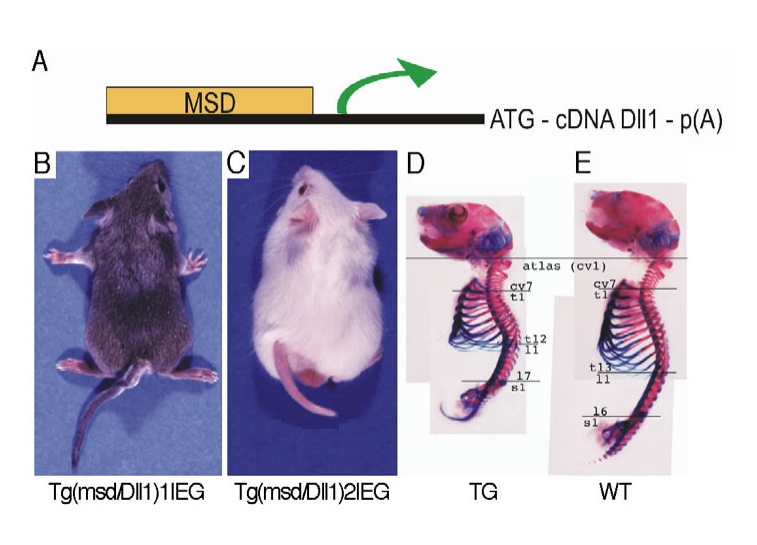
**Mouse line Tg(msd/Dll1)Ieg exhibits a transgenic external and internal phenotype**. (**A**) Scheme of the Tg(msd/Dll1)Ieg transgene vector. (**B-C**) External phenotype of transgenic mice of (**B**) line Tg(msd/Dll1)1Ieg and (**C**) line Tg(msd/Dll1)2Ieg. Both lines show kinked tails and reduced axial length. Skeletal preparations of (**D**) transgenic and (**E**) wild-type mice stained with alizarin red and alcian blue. Although the body length is reduced in transgenic mice compared to wild-type littermates, the number of vertebral elements from the first cervical to the first sacral vertebral element remains unchanged.

To direct the expression of *Dll1 *throughout somites including rostral somite compartments, a full length *Dll1 *cDNA under the control of the mesodermal specific cis-regulatory element (*msd*) was fused to the *Dll1 *minimal promoter [20] (Fig. 1A). Two independent and stable transgenic lines (Tg(msd/Dll1)1Ieg and Tg(msd/Dll1)2Ieg) were established by pronuclear injection. Transgenic mice showed tails with multiple kinks and a reduction in tail and body length with varying severity (Fig. 1B, C). Both transgenic lines exhibited comparable dysmorphologies of the axial skeleton with varying severity. 25% of the transgenic mice born had an externally visible alteration of the phenotype. 18 transgenic animals without externally visible transgenic phenotypes from both the transgenic lines were examined for morphological changes of the axial skeleton by X-ray imaging (Table 1). Abnormal bony fusions of vertebral bodies were found in the thoracic, lumbar and tail regions in 9 out of the 18 transgenic animals. Vertebrae with reduced rostro-caudal length in the thoracic or tail regions were evident in 7 out of the 18 transgenic mice without externally visible phenotype. In 6 out of the 18 animals the number of thoracic and lumbar vertebrae was altered as compared to wild-type littermates and in 1 out of the 18 animals the number of sacral vertebrae was changed. Neural arches were irregular (lacking the spinous process or without bony fusion in the midline) in 11 out of the 18 transgenic mice without externally visible phenotype changes. We did not find dysmorphologies of the vertebral column by X-ray imaging in one of the 18 transgenic animals. Bones of limbs and skull were not affected in any of the transgenic animals. The shortening of the vertebral axis in adult transgenic mice with severe transgenic phenotype was due to frequent fusions of vertebral bodies from thoracic to caudal regions and a reduction of the R/C length of vertebrae caudal to the cervical region (Fig. 1D, E). Thus, the *Dll1 *overexpression phenotype was characterized by various dysmorphologies of the axial skeleton (analysed in detail below) with high penetrance but varying severity.

**Table 1:** X-ray analyses of the axial skeleton of 18 adult mice without an external phenotype from Tg(msd/Dll1)Ieg lines.

number of vertebrae					vertebral fusions					reduced vertebrae				
cv	th	lu	sa	ta	cv	th	lu	sa	ta	cv	th	lu	sa	ta
7	13	6	4	>25	-	-	5–6	-	-	-	-	-	-	2–4
7	**12**	**7**	4	>25	-	-	3–6	4+	+1–4	-	11–12	-	-	-
7	13	6	4	>25	-	-	-	-	-	-	-	-	-	2–3
7	13	6	4	>25	-	-	-	-	-	-	-	-	-	-
7	**12**	**7**	4	>25	-	-	-	-	-	-	-	-	-	-
7	13	6	4	>25	-	13+	+1; 5–6	-	-	-	-	-	-	-
7	13	6	4	>25	-	-	-	-	-	-	-	-	-	-
7	**12**	**7**	**5**	>25	-	-	1–2; 4–5	-	-	-	11–12	-	-	-
7	13	6	4	>25	-	-	-	-	-	-	-	-	-	-
7	**14**	**5**	4	>25	-	-	-	-	-	-	-	-	-	-
7	13	6	4	>25	-	-	3–4	-	-	-	-	-	-	2–4
7	**12**	**7**	4	>25	-	-	4–7	-	-	-	10–12	-	-	-
7	13	6	4	>25	-	-	2–4	-	-	-	-	-	-	-
7	13	6	4	>25	-	-	-	4+	+1	-	-	-	-	-
7	13	6	4	>25	-	-	-	-	-	-	-	-	-	-
7	13	6	4	>25	-	-	-	-	-	-	11–14	-	-	-
7	13	6	4	>25	-	-	-	-	-	-	-	-	-	-
7	**12**	**7**	4	>25		12+	+1	4+	+1	-	-	-	-	-

7	13	6	4	>25	-	-	-	-	-	-	-	-	-	-

cv, cervical; lu, lumbar; ta, tail; th, thoracic; sa, sacral

Bold values differ from the wild-type phenotype presented in the bottom row. Numbers with a '+' indicate fusions of vertebral bodies to the next or previous vertebra. "Reduced vertebra" refers to a shortened R/C length of vertebrae.

### Ectopic *Dll1 *expression throughout somite epithelia

**Figure 2:**
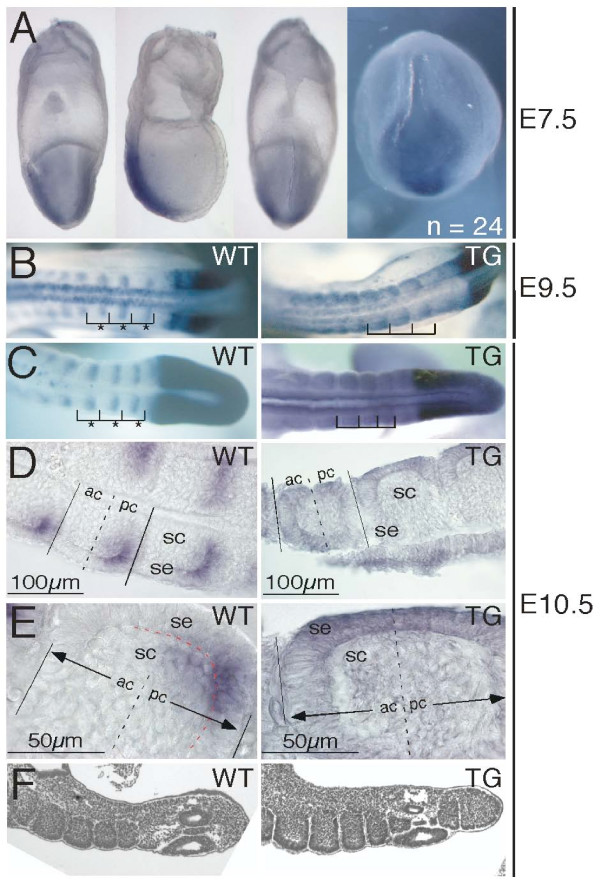
***Dll1 *whole-mount RNA in situ hybridisations and somite formation**. (**A**) *Dll1 *whole mount in situ hybridisations of E7.5 embryos from crosses of transgenic and wild-type mice. 24 embryos were analysed for *Dll1 *expression patterns. We did not observe differences in *Dll1 *expression patterns in these embryos. From left to right: view from the cephalic side, lateral view with the cephalic region to the left and the primitive streak side to the right, view from the side of the primitive streak, view onto the node with the primitive streak on top of the node. Each picture was taken from a different embryo. Ectopic *Dll1 *expression is detected at (**B**) E9.5 and (**C**) E10.5 throughout somites in transgenic embryos. Wild-type expression is restricted to caudal somite halves (asterisks). (**D, E**) Cryo-sections of in situ hybridised E10.5 wild-type embryos reveal expression of *Dll1 *in the caudal, inner epithelium. A weak staining may be present in cells of the caudal somitocoele. The red broken line in the left panel of (**E**) indicates the boundary between the somitic epithelium and the inner somitocoele. Transgenic embryos express *Dll1 *in epithelial cells without caudal restriction. Again, weaker staining is detected in the somitocoele. (**F**) H/E stained sections of E10.5 embryos reveal normal epithelial somites in wild-type and transgenic embryos. ac, anterior compartment of somite; pc, posterior compartment of somite; sc, somitocoele; se, somitic epithelium; TG, transgenic; WT, wild-type.

*Dll1 *expression in wild-type and transgenic mouse embryos was examined from E7.5 to E10.5 by whole-mount RNA in situ hybridisation. At E7.5 no differences between wild-type and transgenic embryos were observed (Fig. 2A). Ectopic *Dll1 *expression in rostral halves of recently formed somites was first detected at E8.0 in transgenic embryos when somitogenesis is initiated and has lasted at least until E10.5 (Fig. 2B to 2E). Variations in the expression level of the transgene were observed but did neither correlate with one of the transgenic lines nor with a specific stage of development. Embryos from whole-mount in situ hybridisations were used for histological sections to determine *Dll1 *expressing cells. In wild-type embryos at E10.5, sections revealed significant staining in cells of the somitic epithelium of caudal somite halves (Fig. 2D, E). A weak staining was detected in the somitocoele adjacent to the caudal epithelium. In transgenic embryos, *Dll1 *expression was also detected in epithelial cells but without restriction to caudal somite compartments (Fig. 2D, E). Similar to wild-type embryos a weak staining was detected in the somitocoele.

### Epithelial somites appear normal in *Dll1 *over-expressing embryos

Histological sections of paraffin embedded embryos at E10.5 revealed no differences between somites of wild-type and transgenic embryos (Fig. 2F). Nascent somites developed a normal epithelial layer surrounding the somitocoele. The size of somites was identical in five age-matched pairs of transgenic and wild-type embryos between E9.5 and E10.5 in raster electron microscopic images (data not shown). Thus, the over-expression of *Dll1 *in the paraxial mesoderm did not affect the early generation of somites with regards to epithelialisation, size and histological appearance at least until E10.5.

**Figure 3:**
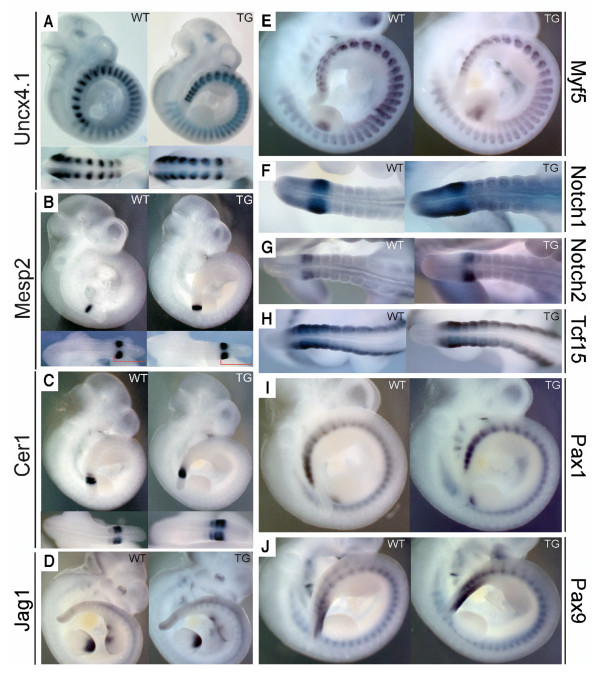
**Expression of *Dll1 *target and cranial and caudal somite marker genes at E10.5 in Tg(msd/Dll1)Ieg transgenic and wild-type embryos**. Top panels in **A **to **C **and panels **D**, **E**, **I**, and **J **show right lateral views of in situ hybridised whole embryos. Lower panels in **A **to **C **and panels **F**, **G**, and **H **show dorsal views of the posterior tail regions. The red brackets in the lower panels of (**B**) indicate the region of the unsegmented, presomitic mesoderm. The gene transcripts to which the in situ probes were specific are indicated next to each panel. We did not detected reproducible changes in gene expression patterns between wild-type and transgenic embryos for the indicated genes at E10.5. TG, transgenic: WT, wild-type.

We analysed the expression of various rostral and caudal somite marker genes and *Notch *signalling targets by whole-mount in situ hybridisation of wild-type and transgenic embryos. From E9.5 to E10.5 we found no differences in the expression levels of *Dll3*, *Jag1*, *Notch1*, *Notch2*, *Lfng*, *Uncx4.1*, *Hes5*, *Mesp1*, *Mesp2*, *Paraxis *(*Tcf15*), *Pax1*, *Pax9*, *Myf5*, *Epha4*, *Myog *and *Cer1 *(Fig. 3A to 3J, and data not shown) [13,21-25]. The R/C marker and *Notch *pathway genes *Notch1*, *Notch2*, *Mesp1*, *Mesp2*, *Myf5*, *Uncx4.1*, *Pax1*, and *Pax9 *were analysed until E12.5 and did not reveal differential gene expression between transgenic and wild-type embryos (Additional file 1). Many of these genes also have dynamic patterns of expression in the PSM. In accordance with the normal and regular epithelial somites observed, we did not find evidence for a change in phased gene expression in the PSM of transgenic embryos in comparison to their wild-type littermates. In particular, the cyclic expression of *Lfng *in the PSM [26,27] was not affected in Tg(msd/Dll1)Ieg embryos. Distinct phases of *Lfng *expression were evident in transgenic embryos at E10.5 (Additional file 2). Homozygous Tg(msd/Dll1)Ieg mice were generated. Dysmorphologies of the axial skeleton of these mice were not different from the dysmorphologies that were identified in heterozygous transgenic mice. We did, therefore, not undertake a gene expression analysis of homozygous transgenic embryos. We cannot exclude that even higher doses of ectopic *Dll1 *signal in the PSM may lead to a different phenotype with regards to cycling gene expression and the generation of R/C polarized epithelial somites.

### *Dll1 *over-expression affects intervertebral articulations and vertebrae morphology

**Figure 4:**
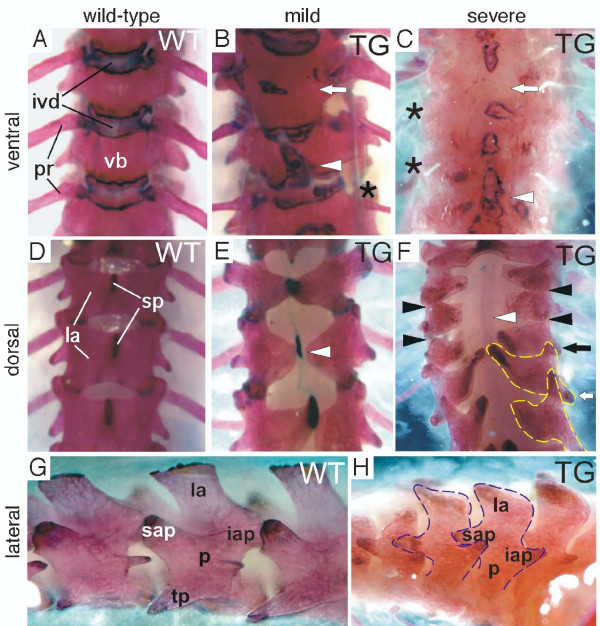
**Alizarin red and alcian blue stained adult skeletons**. Alizarin red and alcian blue stained (**A-F**) thoracic and (**G, H**) lumbar vertebrae. (**B, C**) Split vertebrae (arrowhead), fused vertebrae (arrow) and reduced costal heads of ribs (asterisks) compared to (**A**) wild-type animals are found. (**D-F**) Dorsal examinations exhibit reduced spinous processes, open neural arches (white arrowheads) and reduced (black arrowheads) or malformed (black and white arrows) intervertebral joints. (**G, H**) Reduced intervertebral joints occur in (**H**) transgenic mice compared to (**G**) wild-types but no fused adjacent neural arches (dashed lines) are observed. iap, inferior articular process; ivd, inter-vertebral disc; la, lamina; p, pedicle; pr, proximal rib; sp, spinous process; sap, superior articular process; TG, transgenic; tp, transverse process; vb, vertebral body; WT, wild-type.

Since the contribution of distinct somitic regions to the elements of the axial skeleton has been rather well described [1,17,28,29], we examined vertebrae morphology with the aim to identify those sclerotome and arthrotome derived cell types that contribute to malformed skeletal elements in *Dll1 *transgenic mice. In alizarin red and alcian blue stained skeletons of transgenic mice we observed that structures derived from the somitocoele were frequently missing (Fig. 4). In particular, costal heads of ribs were absent or strongly reduced in their thickness (Fig. 4B, C; asterisks) and IVDs were often missing resulting in the fusion of vertebral bodies (Fig. 4B, C; arrows). The articular processes of the neural arches were either missing in transgenic mice with strong phenotype (Fig. 4F, black arrowheads) or reduced and malformed (Fig. 4F; black and white arrows). Despite the dysmorphologies of articular processes we did not observe fusions between adjacent neural arches even in transgenic animals with severe phenotype (Fig. 4F, H).

Additional dysmorphologies in the axial skeleton of transgenic mice included split vertebral bodies with ectopic pseudo-growth plates (Fig. 4B, C; arrowheads) and open neural arches with missing spinous processes (Fig. 4E, F; white arrowheads). Pedicles and laminae of neural arches were always present and did not fuse between adjacent vertebrae (Fig. 4H).

### Developmental progression of axial dysmorphologies

**Figure 5:**
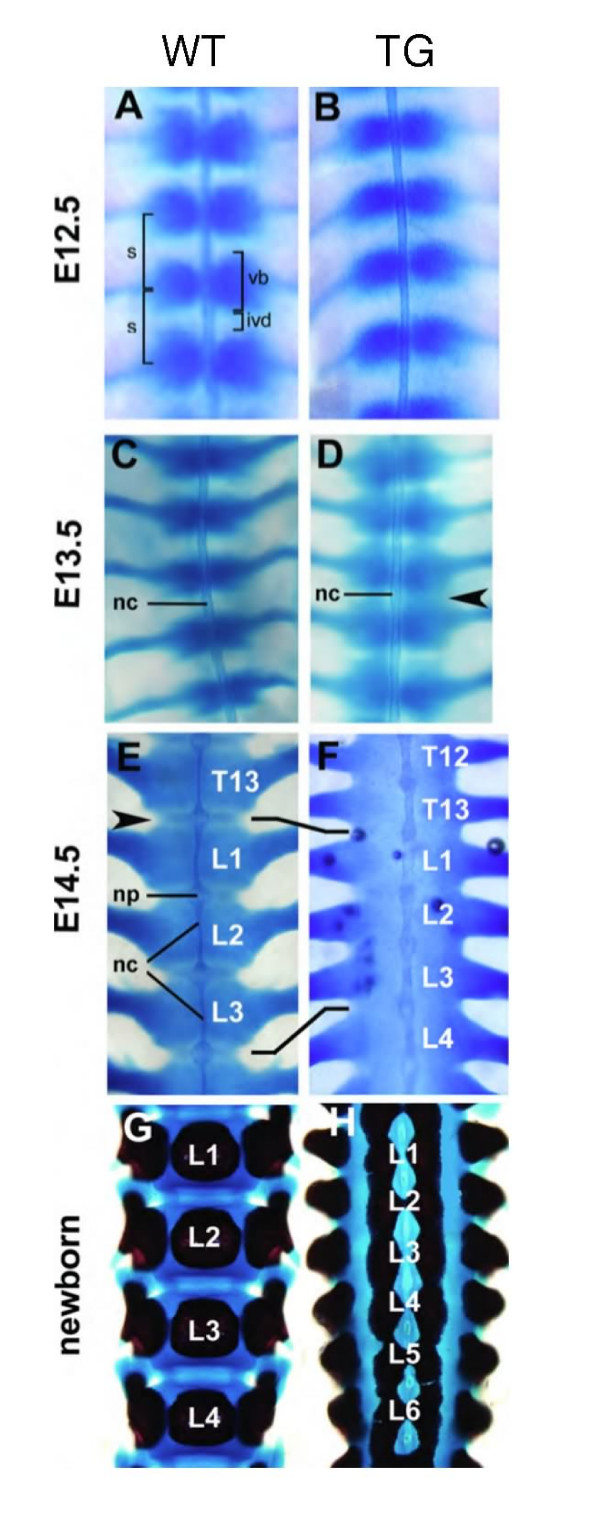
**Developmental progression of the transgenic phenotype in the vertebral column**. (**A-H**) Alizarin red and alcian blue stained wild-type and transgenic lumbar vertebrae, ventral view. (**A, B**) E12.5 reveals no differences between wild-type and transgenic embryos. (**D**) At E13.5 presumptive IVDs stain with alcian blue in transgenic embryos (arrowhead) but not in (**C**) wild-types. At E14.5 (**E**) vertebrae of wild-type embryos are separated by weaker stained IVDs (arrowhead). The notochord is extruded to the nucleus pulposus. (**F**) Transgenic vertebrae are shortened, stain uniformly with alcian blue, IVDs are missing and the notochord remains as a rod like structure. Newborn transgenic mice (**H**) exhibit two lateral centres of ossification compared to one centre in wild-type mice (**G**). ivd, intervertebral disc; L, lumbar vertebra; nc, notochord; np, nucleus pulposus; s, somite; T, thoracic vertebra; TG, transgenic mouse; vb, vertebral body; WT, wild-type.

To further characterise the loss of intervertebral joints and midline clefts of the axial skeleton, the developmental progression of this transgenic phenotype was monitored from E12.5 to the newborn stage using alcian blue and alizarin red stainings (Fig. 5). Pairwise chondrocyte condensations of the presumptive axial skeleton adjacent to the notochord appeared normal in transgenic embryos at E12.5 compared to wild-type littermates (Fig. 5A, B). In wild-type embryos at E13.5 the pairs of cell condensations have come into contact at the axial midline. Here they now form single, rather cylindrical cell condensations that periodically encircle the notochord (Fig. 5C). In transgenic embryos at E13.5 pairs of cartilage condensations were not yet merged at the axial midline and regions between successive presumptive vertebrae stained with alcian blue (arrowhead in Fig. 5D). In wild-type embryos at E14.5 morphogenesis had proceeded and a regular pattern of strongly alcian blue stained presumptive vertebral bodies and weaker stained presumptive IVDs was evident (Fig. 5E). Contrary, in transgenic embryos of the same age, the developing vertebral column stained uniformly with alcian blue (Fig. 5F). The extrusion of notochordal cells in transgenic embryos was incomplete such that the rod of axial mesoderm in the regions of vertebral bodies was thicker than in wild-type embryos (Fig. 5E, F). In vertebral bodies of newborn wild-type mice, alizarin red staining showed a single, central core of mineralised extracellular matrix (Fig. 5G). Mineralisation had progressed also in the early neural arches and morphogenesis had resulted in the formation of superior and inferior articular processes forming the intervertebral joints. In contrast, transgenic newborn mice showed two mineralised cores lateral to the axial midline in each vertebral body (Fig. 5H). Intervertebral discs were missing and adjacent vertebral bodies fused. Superior and inferior articular processes were missing in the newborn transgenic mice with severe phenotype (Fig. 5H). Thus, the loss of IVDs and the failure of sclerotome cells to merge at the axial midline was traced back as early as E13.5 in transgenic embryos.

In addition to altered cell differentiation of presumptive IVD cells in transgenic mice, we considered the possibility that the loss of IVD cells might result either from apoptosis of presumptive IVD cells or, alternatively, from over-proliferation of cells of the prospective vertebrae displacing future IVD cells. To analyze these alternatives, we performed Bromodeoxyuridine (BrdU) and TUNEL assays (BrdU: E12.5 (n = 4), E13.5 (n = 4); TUNEL: E12.5 (n = 8), E13.5 (n = 3), E14.5 (n = 6), E15.5 (n = 4) and E17.5 (n = 3)). No significant differences between wild-type and transgenic animals were observed in BrdU (Additional file 3) and in TUNEL assays (Additional file 4).

### *Dll1 *over-expression affects chondrocyte hypertrophy and endochondral bone formation in vertebrae

**Figure 6:**
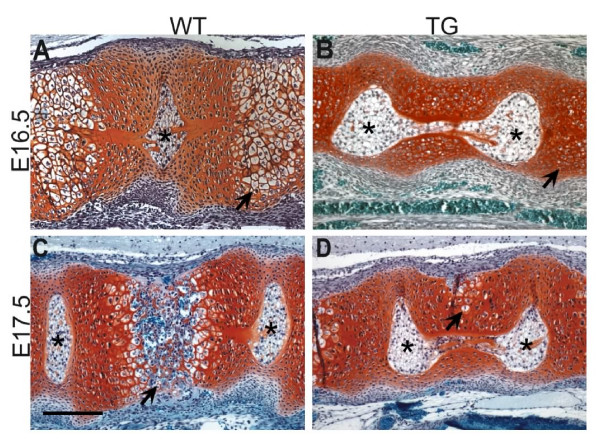
**Safranin O stainings for cartilage of mid-sagittal sections through the presumptive vertebrae of transgenic and wild-type embryos**. (**A**) In wild-type embryos at E16.5 hypertrophic chondrocytes are observed in the centre of the vertebrae (arrow). (**B**) The number of chondrocytes undergoing hypertrophic differentiation is reduced in transgenic embryos (arrow). At E17.5 (**C**) chondrocytes of wild-type embryos lose the cartilage matrix and vascularisation and invasion of osteocytes starts (arrow). The notochord is extruded from the vertebrae. (**D**) In transgenic vertebrae of the same age fewer cells are hypertrophic and are dorsally displaced (arrow). Ossification is delayed and notochord extrusion is incomplete. Scale bar: 200 μm. Asterisks: nucleus pulposus.

We performed Safranin O stainings on histological sections of paraffin embedded embryos to investigate cartilage development in prospective vertebral bodies (Fig. 6). These stainings revealed fewer hypertrophic chondrocytes in vertebral bodies of transgenic rather than of wild-type embryos (E16.5) (Fig. 6A, B; arrows). Ossification had progressed normally at E17.5 in wild-type animals but was delayed in transgenic embryos (Fig. 6C, D; arrows) and hypertrophic chondrocytes were dorsally displaced (Fig. 6D; arrow). RNA in situ hybridisations to detect *Col10a1 *gene expression, which is a marker for cells undergoing endochondral ossification [30,31], also revealed fewer hypertrophic cells (data not shown).

## Discussion

Mouse *Dll1 *expression in the PSM and caudal somite halves, is necessary for the formation of epithelial somites and maintenance of caudal somite identity [32,33]. We established two transgenic mouse lines (Tg(msd/Dll1)Ieg) over-expressing *Dll1 *in the paraxial mesoderm including rostral somite compartments under the control of the msd cis-regulatory element [34,35]. We investigated the role of restricted, compartmentalised *Dll1 *expression for the maintenance of R/C somite identity and the morphogenesis of the axial skeleton. Tg(msd/Dll1)Ieg animals were viable and fertile. They developed normal epithelial somites and gene expression analysis on R/C marker genes revealed, that the uniform over-expression of *Dll1 *throughout the somites was not sufficient to confer caudal compartment identity to rostral somite halves. Nevertheless transgenic animals had at least three distinct phenotypic alterations of the vertebral column: (i) arthrotome related dysmorphologies, (ii) midline defects and (iii) a failure in chondrocyte maturation. In the following, we discuss the potential role of *Dll1 *over-expression for these phenotypic traits and the requirement of caudally restricted *Dll1 *expression for intervertebral joint formation.

### Somite formation and rostro-caudal polarity are not affected by *Dll1 *over-expression

*Dll1 *deficient embryos (*Dll1*^*tm*1*Gos*/*tm*1*Gos*^) showed severe patterning defects in the paraxial mesoderm and died before E12.5 [33]. In these mutants, the R/C pattern of somites was not established and maintained as in wild-type embryos and nascent somites did not epithelialise normally. The requirement of *Dll1 *for the formation of the axial skeleton was also studied in a mouse model expressing a truncated and dominant-negative version of *Dll1 *(*Dll1*^*dn*^) under the control of the msd cis-regulatory element (Tg(msd/Dll1^*dn*^)) [35]. In the axial skeleton such transgenic mice exhibited fusions of laminae of neural arches, reduction or loss of pedicles, split vertebral bodies as well as rostral homeotic transformations at the cervical-thoracic transition. Gene expression analyses revealed a reduction of *Uncx4.1 *expression in caudal compartments and an expansion of the expression domain of the rostral somite marker gene *Tbx18*. These data suggested a partial loss of caudal somite compartment characteristics in Tg(msd/Dll1^*dn*^) animals. Despite the fact that Tg(msd/Dll1)Ieg animals exhibited dysmorphologies of the axial skeleton, *Dll1 *transgenic embryos did not display changes in R/C marker gene expression.

The midline defects in the axial skeleton, the vertebral fusions and dysmorphologies of ribs observed in Tg(msd/Dll1)Ieg animals were reminiscent of somitogenesis related phenotypes observed in other mutant mice. All of these mutant animals are, however, characterised by alterations of R/C marker gene expression. For example, the bilateral centres of ossification in the vertebral bodies of *Paraxis *[36-39], *Dll3 *[40-42] and *Psen1 *[14,25,43,44] deficient mice as well as missing spinous processes in *Uncx4.1*^-/- ^[23,24] and *Dll3 *deficient skeletons resemble the midline defects found in the *Dll1 *over-expressing mice. Proximal rib malformations and/or rib fusions occur consistently in *Paraxis*, *Psen1, Dll3 *and *Uncx4.1 *as well as in the *Dll1 *over-expressing transgenic mice. Fusions of vertebral bodies or precursors along the R/C axis and the loss of IVDs as described for Tg(msd/Dll1)Ieg animals were also found in *Paraxis*, *Psen1 *and occasionally in *Uncx4.1 *deficient mice. Our analysis of the Tg(msd/Dll1)Ieg mice clearly shows that these phenotypes can occur in mice with apparently normal R/C polarity of somites. The fact that rostral and caudal sclerotome derived structures were not missing in the Tg(msd/Dll1)Ieg mice additionally supports the hypothesis that R/C somite polarity is not affected and suggests an alternative origin of the axial dysmorphologies. A potential reason for the finding that transgenic *Dll1 *over-expression does not affect the polarity of somites maybe that the endogenous expression of *Dll3 *inhibts the ectopic activation of Notch signalling in anterior somite compartments [45].

### *Dll1 *over-expression might affect arthrotome cell differentiation

In addition to midline defects, we observe the loss or reduction of IVDs, articular (zygapophyseal) joints and proximal ribs in the axial skeletons of Tg(msd/Dll1)Ieg animals. These phenotypic characteristics are present despite normal epithelialisation and R/C polarity in transgenic mice. One possible explanation for the transgenic phenotype affecting the intervertebral joints may be that the loss of restricted *Dll1 *expression results in a late defect in resegmentation of sclerotomal compartments. Alternatively, we noted that all the vertebral structures (IVDs, articular joints, and proximal ribs) that are affected in *Dll1 *over-expressing mice are located in a central position of the vertebral motion segment. Injection of single somite cells with fluorescent dye [46] and homotopical grafting experiments of quail and chicken somitocoele cells [16,17] had previously suggested that somitocoele cells form a distinct somitic compartment, the arthrotome. During later development these cells are located at a central position of the vertebral motion segment forming vertebral joints, IVDs and the proximal ribs [18]. Accordingly, the microsurgical removal of somitocoele cells from chick epithelial somites and preventing the epithelial cells from contributing to the somitocoele cell population, resulted in the loss of IVDs, fusion of vertebral bodies and the absence of intervertebral joints [19]. These analyses on the immediate fate of somitocoele cells have consistently been performed in the avian system [17,46-48]. A related study in mice showed *Pax1 *expression in somitocoele cells and the ventromedial sclerotome. Mice deficient for the *Pax1 *gene lack derivatives of the ventromedial sclerotome or have reduced IVDs, proximal ribs and articular processes [49]. Considering the experimental evidence on the fate of somitocoele cells in the avian embryo and of *Pax1 *expressing cells in mice, we propose the hypothesis that the observed reduction or loss of central structures of the vertebral motion segment may be due to a failure in the development or specification of the arthrotome in Tg(msd/Dll1)Ieg transgenic embryos. However, we did not observe any changes in the expression of *Pax1 *in transgenic embryos. This observation together with the normal histological appearance of somites suggests that the *Dll1 *over-expression throughout somites and the PSM does not affect the initial formation of somitocoele cells. Instead we consider the possibility that the over-expression of *Dll1 *in epithelia of somites might affect the contribution of epithelial cells to the somitocoele [19,46].

### *Dll1 *over-expression negatively regulates chondrocyte differentiation

Tg(msd/Dll1)Ieg animals exhibited a delay in chondrocyte hypertrophy and endochondral bone formation in presumptive vertebral bodies. Mid-sagittal as well as lateral-sagittal sections through the vertebral column of transgenic embryos at E16.5 and E17.5 displayed less hypertophic chondrocytes that undergo endochondral ossification than in wild-type embryos (Fig. 6B, D). Previous *lacZ *reporter gene analyses in *Dll1*^+/*tm*1*Gos *^animals [33] revealed no *Dll1 *expression in presumptive vertebral bodies, IVDs or the notochord [34]. We therefore assume that chondrocyte maturation was inhibited in transgenic embryos due to the earlier sclerotomal over-expression of *Dll1*. These observations essentially confirm previous studies that have revealed that *Delta*/*Notch *signalling acts as a negative regulator for the transition from pre-hypertrophic to hypertrophic chondrocytes [50-52].

## Conclusion

In conclusion, the over-expression of the *Dll1 *gene in rostral epithelial somites was not sufficient to confer caudal somite identity to rostral compartments in transgenic embryos. It had been suggested previously that the compartmentalised expression of *Dll1 *later in epithelial somites might be necessary for the maintenance of segment boundaries. However, it has been unclear so far, through which biological process *Dll1 *could function to maintain segment boundaries. Our data from transgenic mice over-expressing *Dll1 *suggest that the restricted *Dll1 *expression in caudal epithelial somites may be required for the proper development of the arthrotome compartment. The failure of arthrotome development in Tg(msd/Dll1)Ieg mice leads to distinct dysmorphologies of the central region of the vertebral motion segment. The mechanism through which *Dll1 *acts to specify cells of the arthrotome still needs to be elucidated.

## Methods

### Cloning of the Tg(msd/Dll1)Ieg construct and generation of two transgenic mouse lines

The 1.4 kb msd – element was introduced upstream the *Dll1 *minimal promoter as previously described [20,35], followed by a full length *Dll1 *cDNA cloned in frame with the start codon and the SV40 and PGK polyadenylation signals [32]. The construct was isolated as fragment and microinjected into pronuclei of hybrid CByB6 fertilised eggs according to standard procedures. Two independent transgenic lines (Tg(msd/Dll1)1Ieg and Tg(msd/Dll1)2Ieg) were established and maintained on a C3H background.

### Genotyping of transgenic mice

Genomic DNA from yolk sacs or tail biopsies was used for PCR genotyping. With the primers 5'-CGATACCCAGGTTGTCTCC-3' (exon 6) and 5'-AGCACACTCATCTACTTCCAG-3' (exon 7) a 347 bp and a 225 bp PCR product for the wild-type and the transgene were amplified, respectively.

### Skeletal preparations, safranin O and hematoxylin and eosin (H/E) staining

Skeletal preparations of embryos, newborn and adult mice were performed by alizarin red and alcian blue staining according to standard procedures [53]. Safranin O staining on paraffin embedded sections was performed as described previously [54]. H/E staining was performed according to standard procedures [55].

### Whole-mount in situ hybridisation

Whole-mount in situ hybridisation was performed according to standard procedures [56,57]. Embryos were fixed, dehydrated by ascending methanol series, bleached in 14% H_2_O_2 _for one hour and stored in 100% methanol at -20°C. Antisense riboprobes were generated using the DIG-RNA labelling system (Roche, Germany) and hybridisation was performed at 68°C over night. BM Purple AP substrate (Roche, Germany) was used for colour development at 4°C for 24 – 48 hours. After staining was complete embryos were post fixed in 4% PFA. For sections embryos were cryo-protected in 30% sucrose in PBS, OCT embedded, cryo-sectioned at 35 μm and mounted. The following probes were used: *Dll1 *[32]; *Dll3 *[58]; *Jag1 *[59]; *Notch1 *[60]; *Notch2 *[61]; *Lfng *[62]; *Uncx4.1 *[63]; *Hes5 *[64]; *paraxis *[37]; *Mesp2 *[25]; *Pax1 *[65]; *Pax9 *[66]; *Myf5 *; *Mesp1 *[67]; *Epha4*; *Myog*; *mCer*; *Col10a1*.

### Proliferation (BrdU) and apoptosis (TUNEL) assays

Cell proliferation was analysed by detection of incorporated BrdU (Sigma, Germany) in mouse embryos of day 12.5 and 13.5 (E12.5 and E13.5) according to published protocols [24,68]. TUNEL assay was performed as described by Kingsley-Kallesen [69].

## Supplementary Material

Additional File 1 **Whole mount in situ hybridisations of E12.5 wild-type (wt) and transgenic (tg) mouse embryos**. In each panel a representative wt embryo is shown on the left and a transgenic embryo is shown on the right. The upper photograph in each panel shows a whole embryo either in a ventral (**A**) or lateral view (**B**, **C**, and **D**). The photographs below show details from the pre-somitic mesoderm and several pairs of somites; posterior is to the left in the lower photographs. Hybridisation with a probe for *Uncx4.1 *(**A**), *Pax9 *(**B**), *Pax1 *(**C**), and *Notch1 *mRNA (**D**). Click here for file

Additional File 2 **Three phases of cyclic *Lfng *expression in the paraxial mesoderm of Tg(msd/Dll1)Ieg embryos at E10.5**. (**A**) After somite formation a weak expression domain is initiated in the tail bud and the recently formed somite, strong expression is detected in the rostral PSM. (**B**) The strong expression domain is expanded in the PSM and (**C**) *Lfng *is expressed in the whole PSM, a weaker domain marks the line of the next somite formation. Click here for file

Additional File 3 **BrdU assay in frontal (A and C) and mid-sagittal sections (B and D) of E12.5 and E13.5 wild-type (wt, left) and transgenic (tg, right) embryos**. (**A**) shows frontal sections at the level of the notochord (nt) through a wt (left) and transgenic (right) embryo at E12.5. Presumptive regions of intervertebral discs (ivd) and the region of the sclerotome (scl) can be histologically distinguished. There is no difference in the number of BrdU labelled cells if regions of ivd and scl are compared. The sectioned transgenic embryo (right) shown in panel (**B**) has a severe phenotype: There is no clear metameric sequence of ivd and scl regions. At E13.5 (**C **and **D) **the formation of the nucleus pulposus (np) initiates. Also at this stage there is no change in the number of BrdU labelled cells if scl and ivd are compared in wt and tg embryos. Numbers above scale bars indicate the size in μm. Click here for file

Additional File 4 **TUNEL assay and DAPI stainings in sections of E12.5 (A – E), E13.5 (F – K), and E15.5 (L – Q) wild-type (wt) and transgenic (tg) embryos**. Panels (**A **– **D**) show frontal sections at the level of the notochord in the thoracic region. Notochord (nt), sclerotome (scl), and presumptive intervertebral regions (ivd) are histologically recognizable in DAPI stainings (left panels). Based on TUNEL assays (right panels, **B **and **D**) no indication of increased DNA fragmentation was evident in any particular region of the section. Panel (**E**) shows a positive control that was DNase treated. Sagittal sections through the lumbar (**F **– **I**) and sacral (**J**, **K**) regions did not reveal increased fluorescence in particular regions in the TUNEL assay in wt (**F **and **G**) and transgenic (**H **– **K**) embryos at E13.5. Panels (**L **– **O**) show sagittal sections through the thoracic regions of a wt (**L**, **M**) and a transgenic (**N**, **O**) E15.5 embryo. The presumptive vertebral bodies and a future ivd are discernible in the DAPI stainings. No increased fluorescence was detected in these regions in the TUNEL assay. Panel (**Q**) shows a DNase treated positive control for the TUNEL assay and panel (**P**) is a DAPI staining of the same section. Click here for file
