# Supplementary figures and images for: Compartmentalised expression of Delta-like 1 in epithelial somites is required for the formation of intervertebral joints
Source: BMC Dev Biol. 2007 Jun 17;7:68. doi: 10.1186/1471-213X-7-68 (PMC1924847; doi:10.1186/1471-213X-7-68)

Uncx4.1

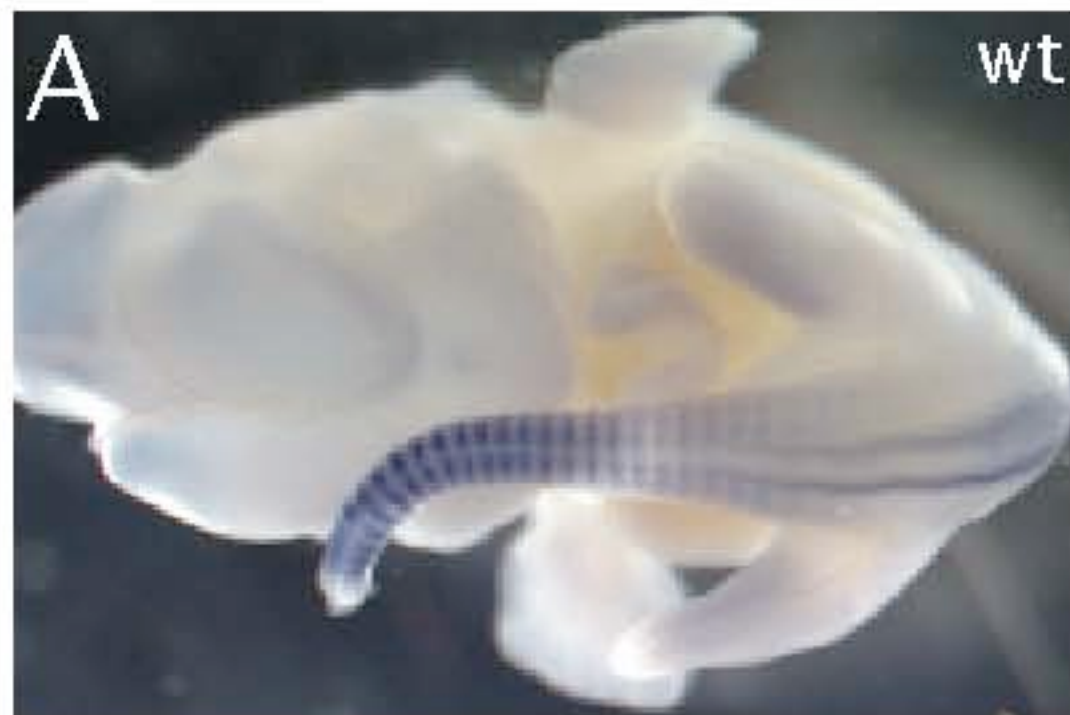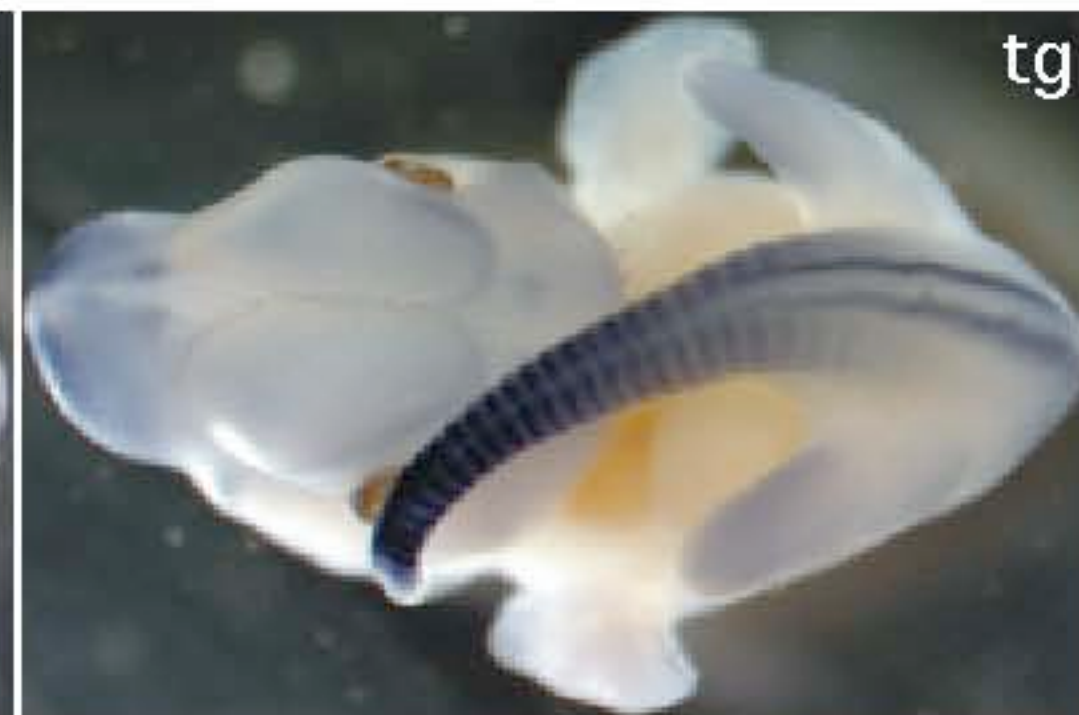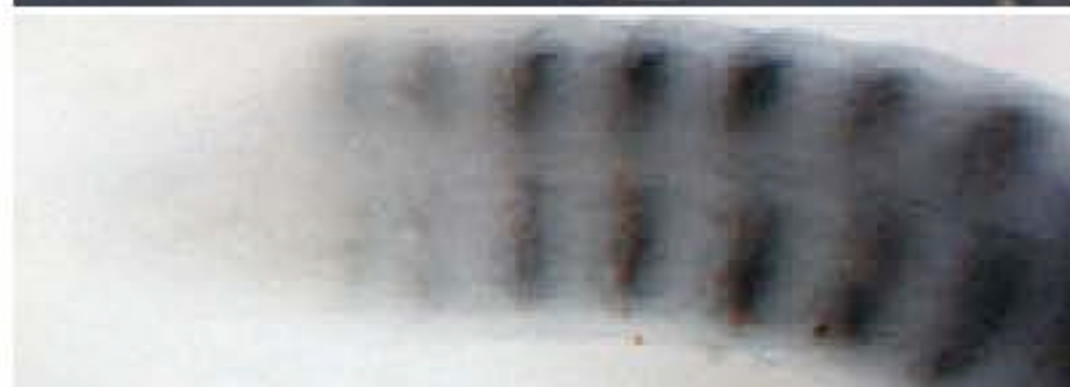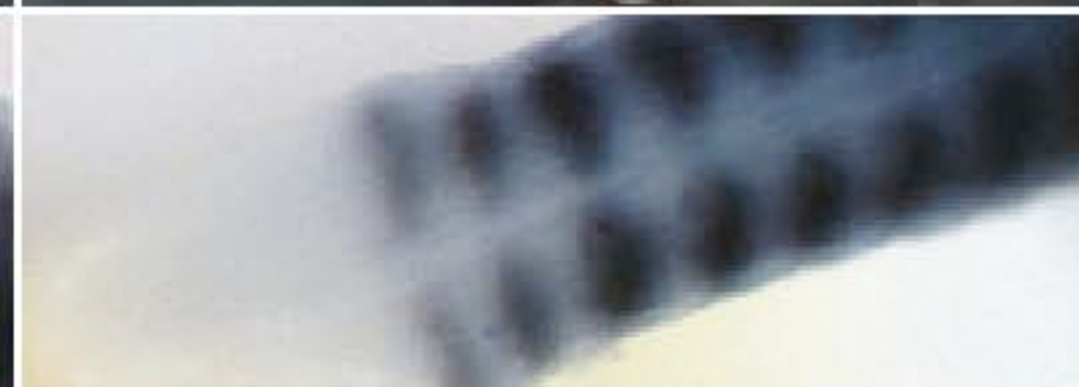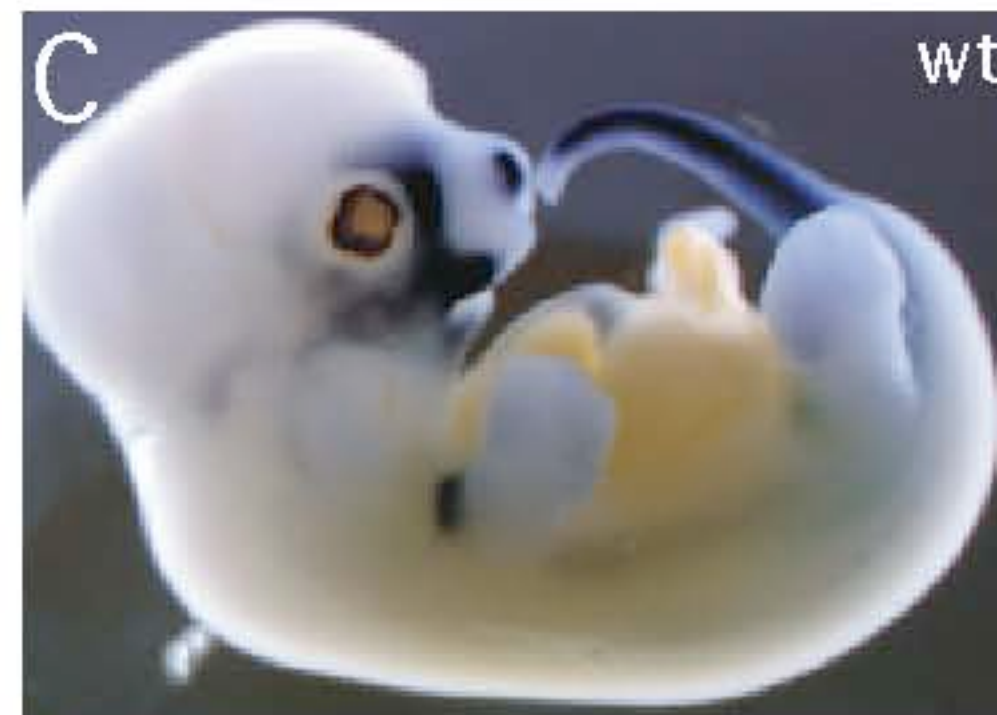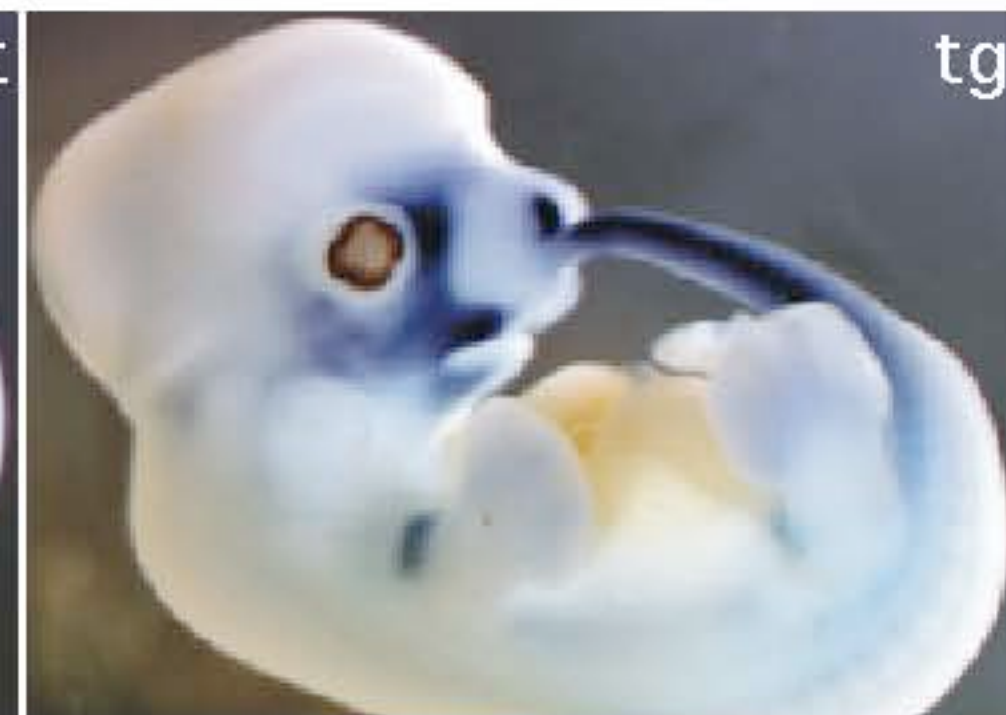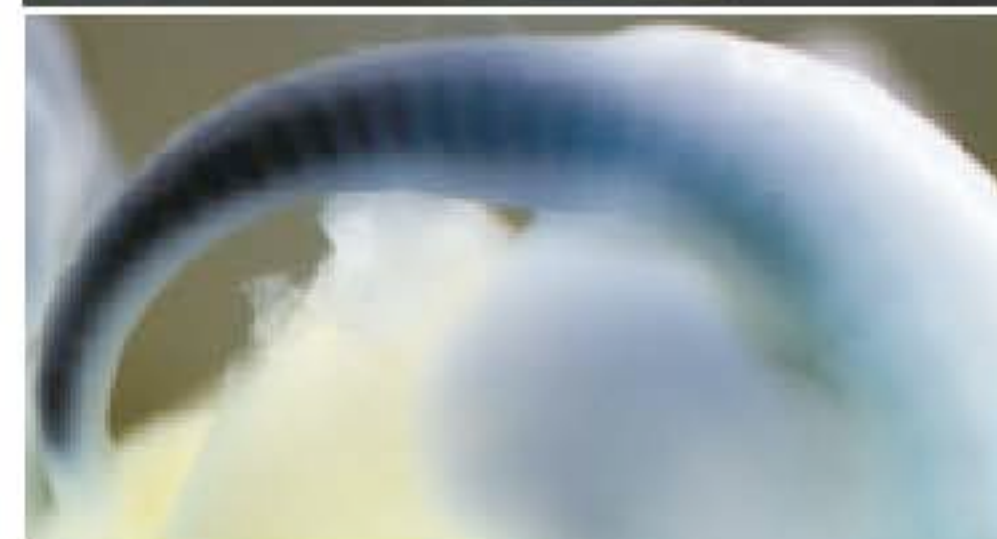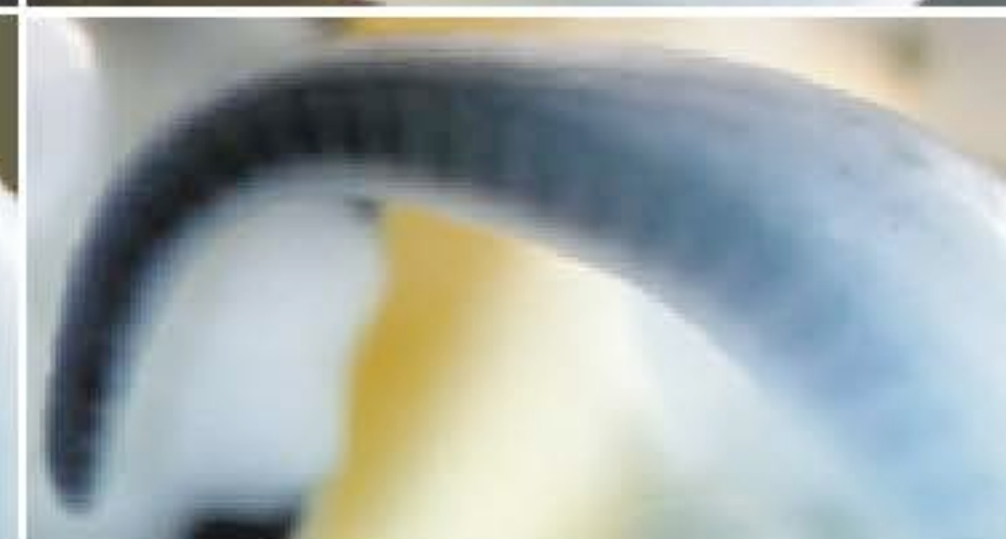

Pax1

Pax9

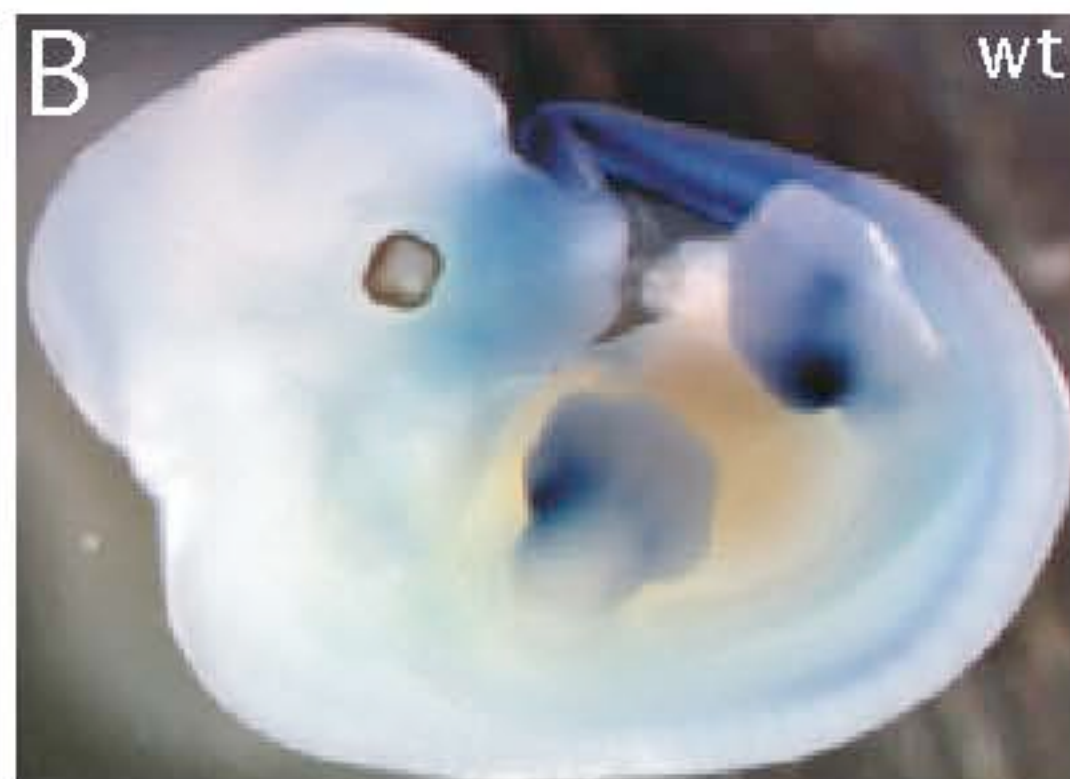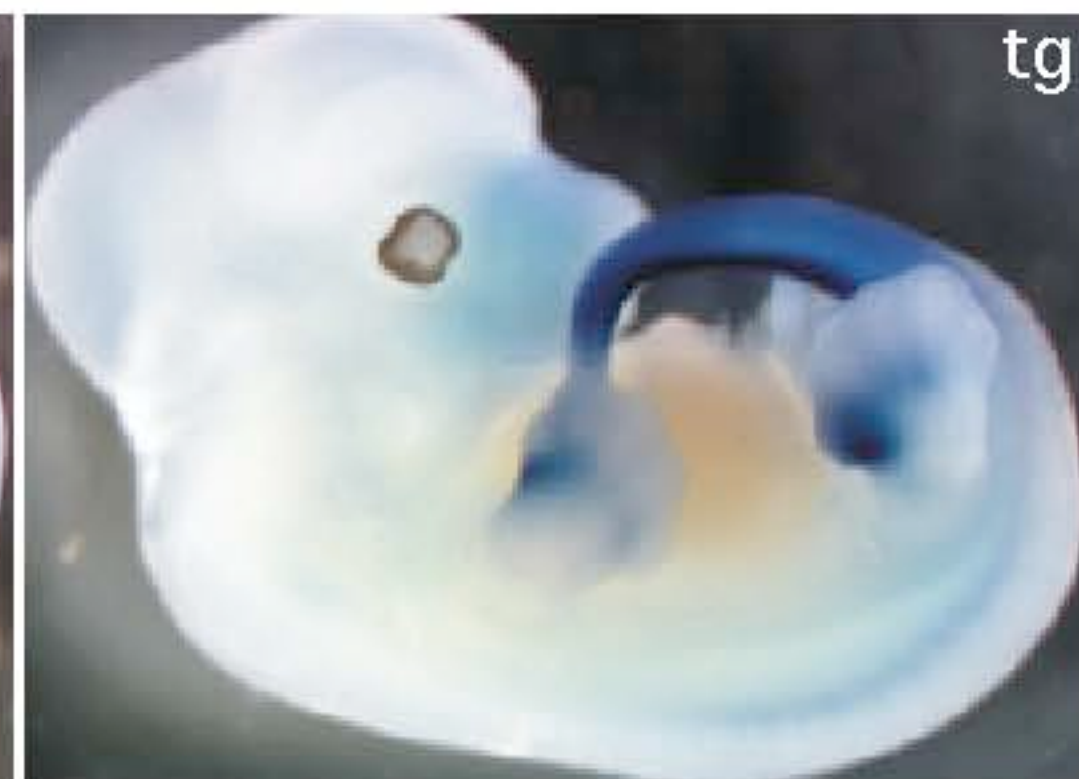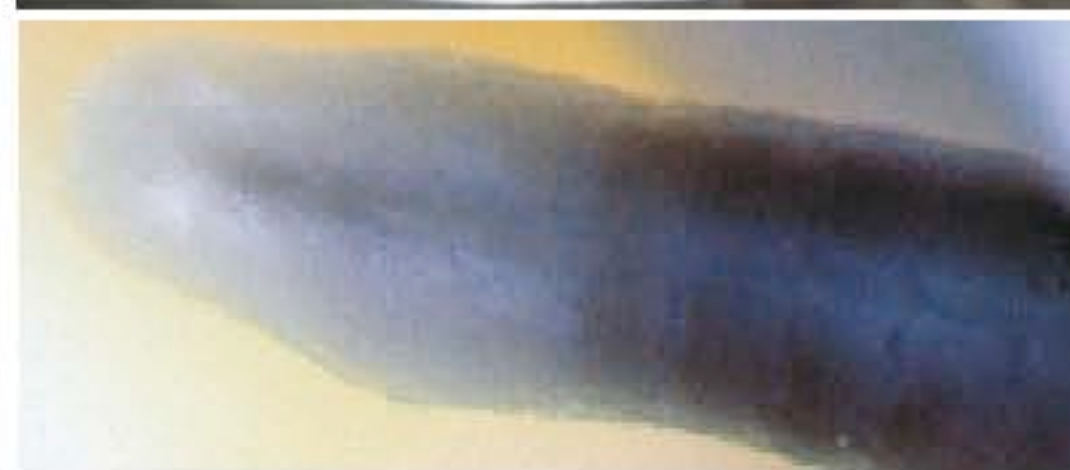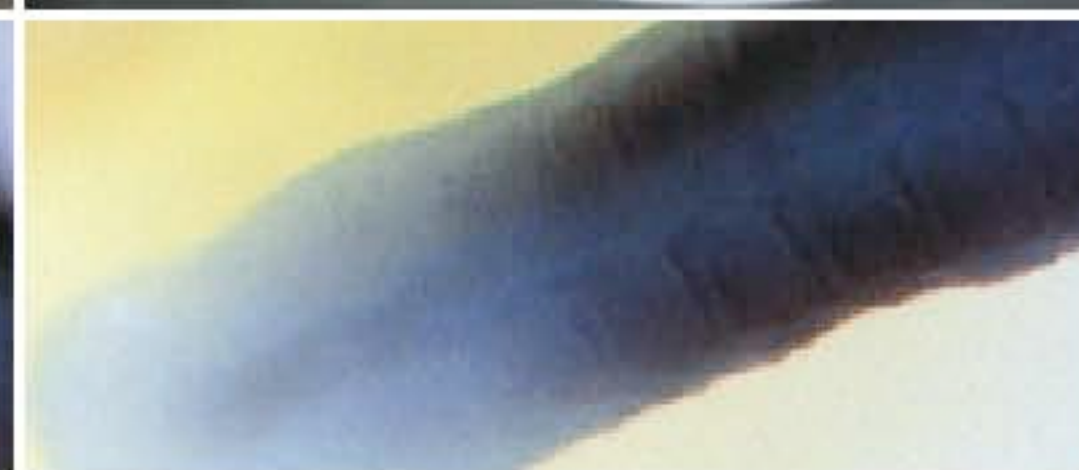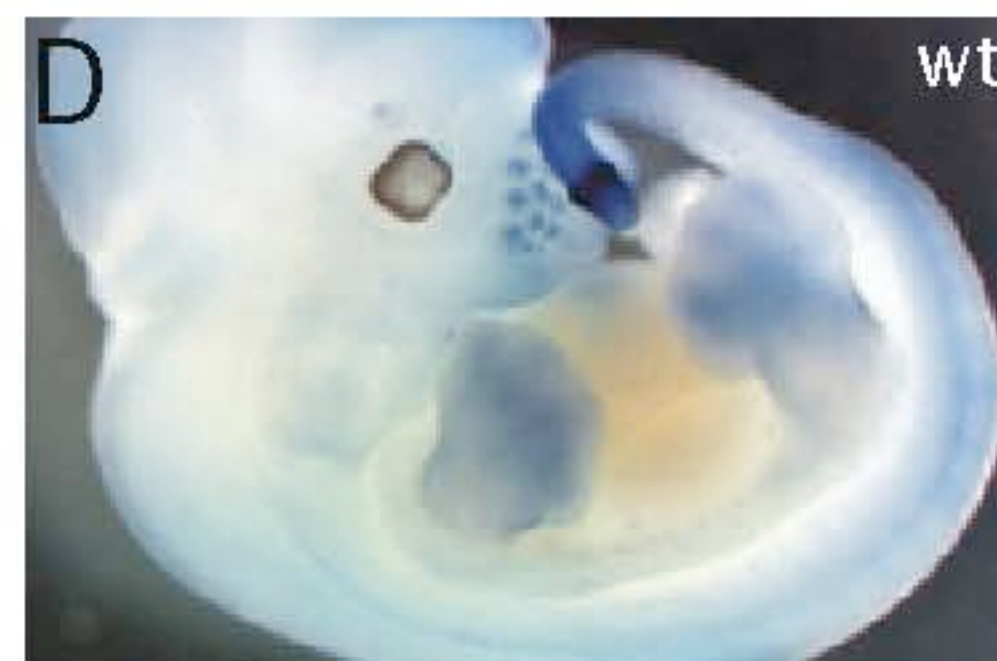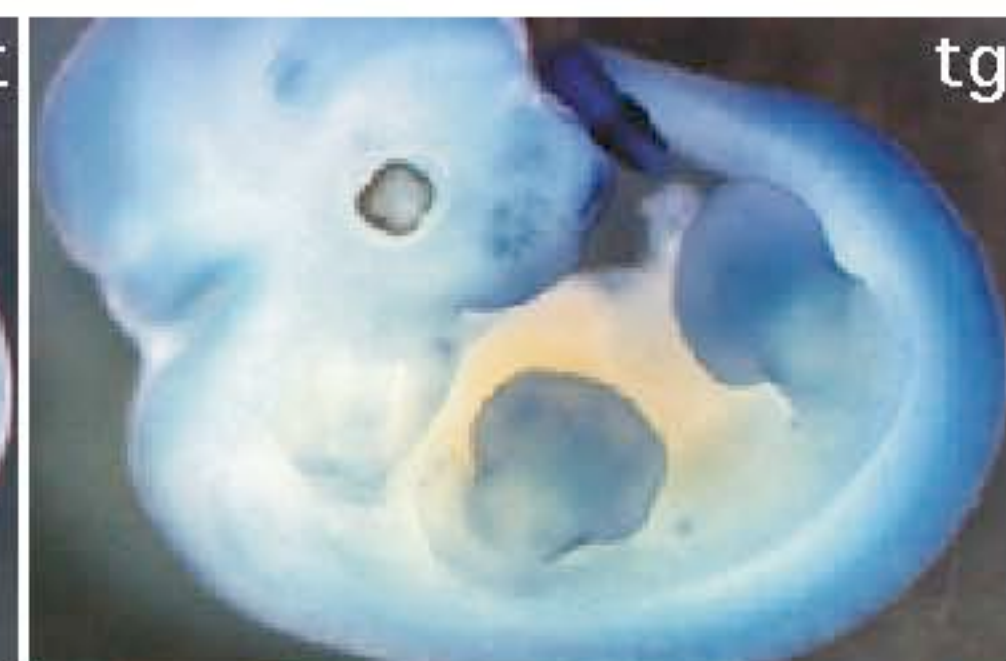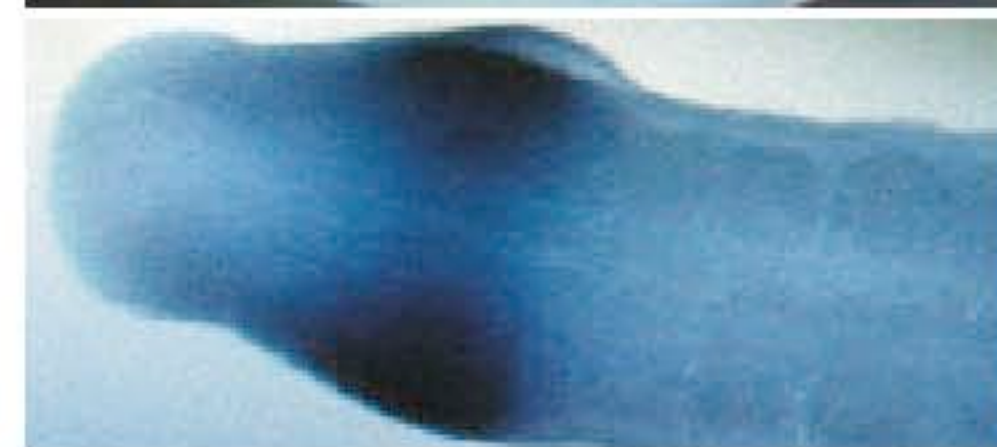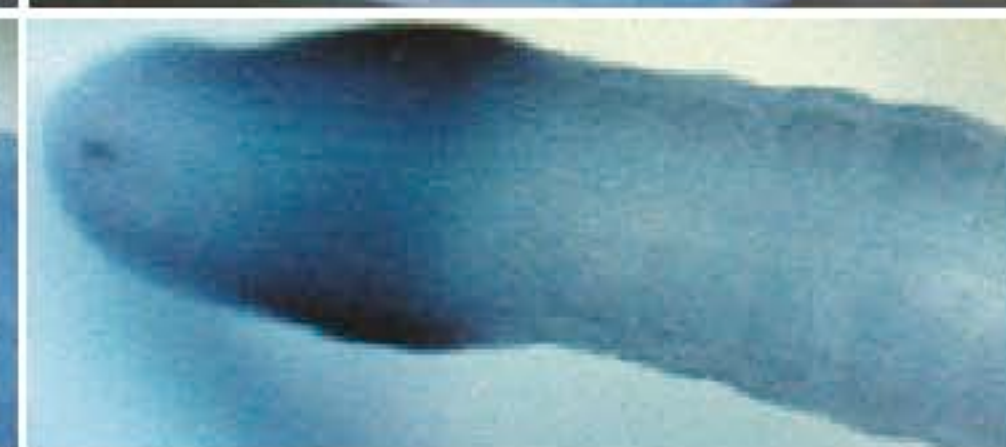

Notch1

Supplement: Additional File 1 — Whole mount in situ hybridisations of E12.5 wild-type (wt) and transgenic (tg) mouse embryos. In each panel a representative wt embryo is shown on the left and a transgenic embryo is shown on the right. The upper photograph in each panel shows a whole embryo either in a ventral (A) or lateral view (B, C, and D). The photographs below show details from the pre-somitic mesoderm and several pairs of somites; posterior is to the left in the lower photographs. Hybridisation with a probe for Uncx4.1 (A), Pax9 (B), Pax1 (C), and Notch1 mRNA (D). [file 1471-213X-7-68-S1.pdf]

**A**

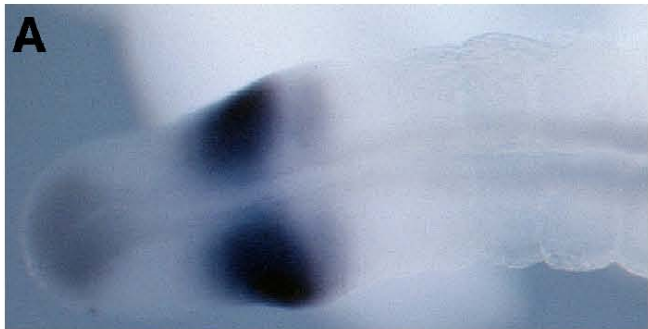

**B**

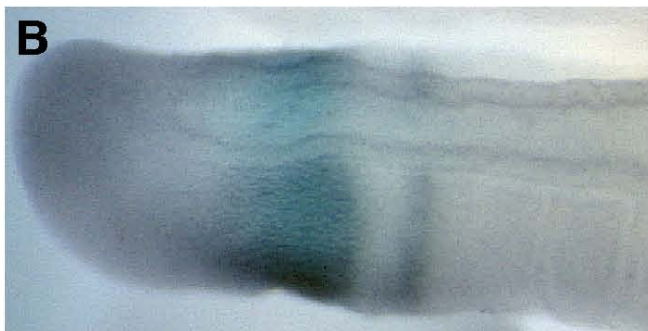

**C**

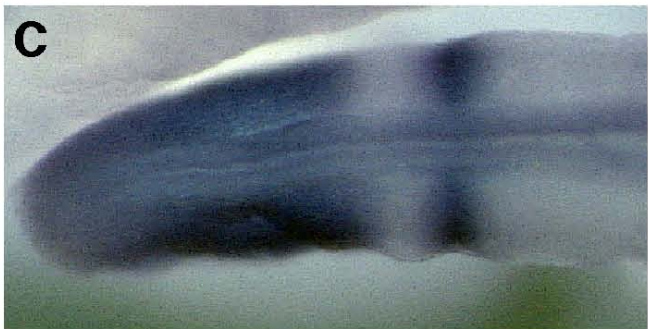

Supplement: Additional File 2 — Three phases of cyclic Lfng expression in the paraxial mesoderm of Tg(msd/Dll1)Ieg embryos at E10.5. (A) After somite formation a weak expression domain is initiated in the tail bud and the recently formed somite, strong expression is detected in the rostral PSM. (B) The strong expression domain is expanded in the PSM and (C) Lfng is expressed in the whole PSM, a weaker domain marks the line of the next somite formation. [file 1471-213X-7-68-S2.pdf]

wt

tg

frontal

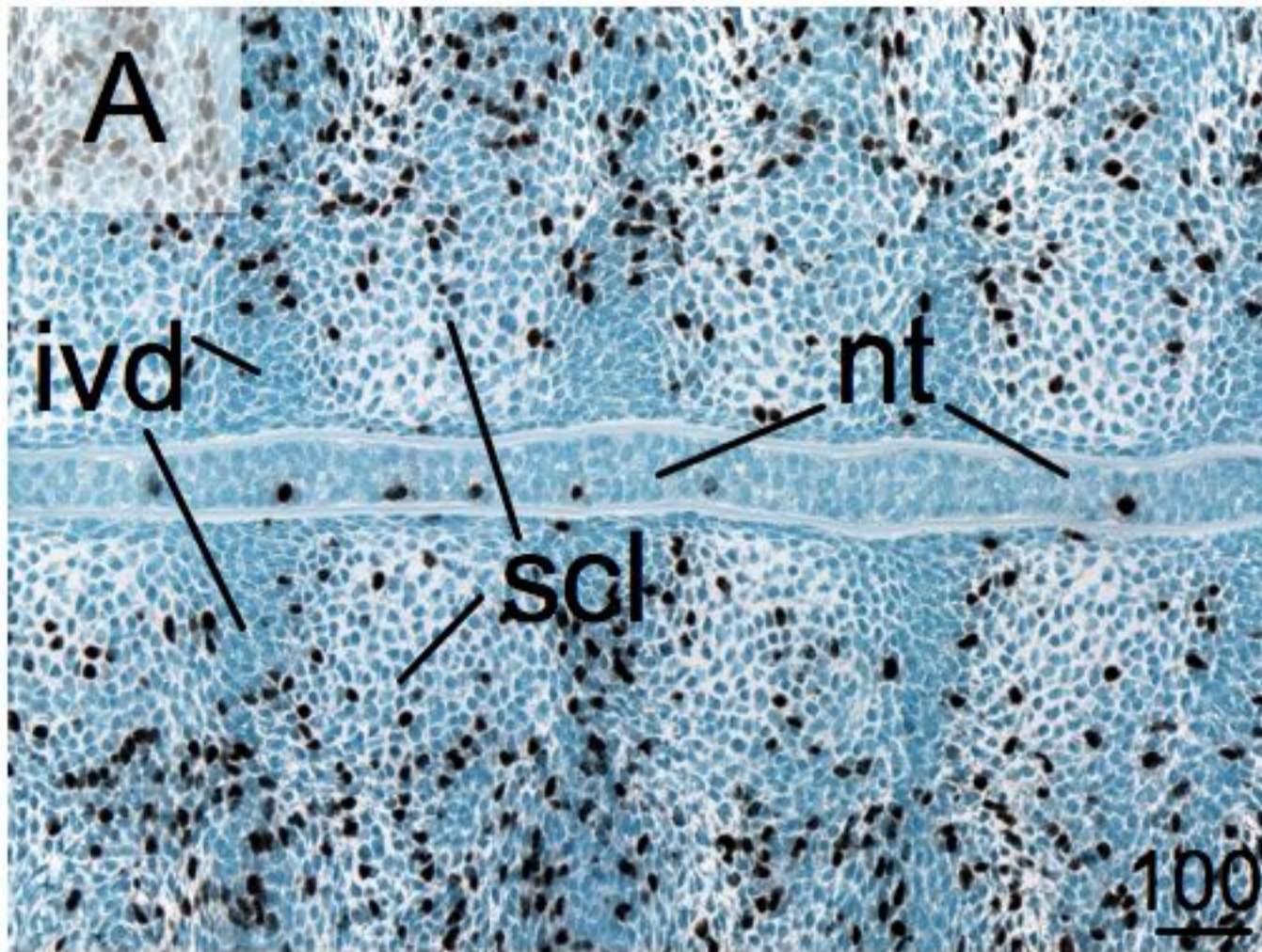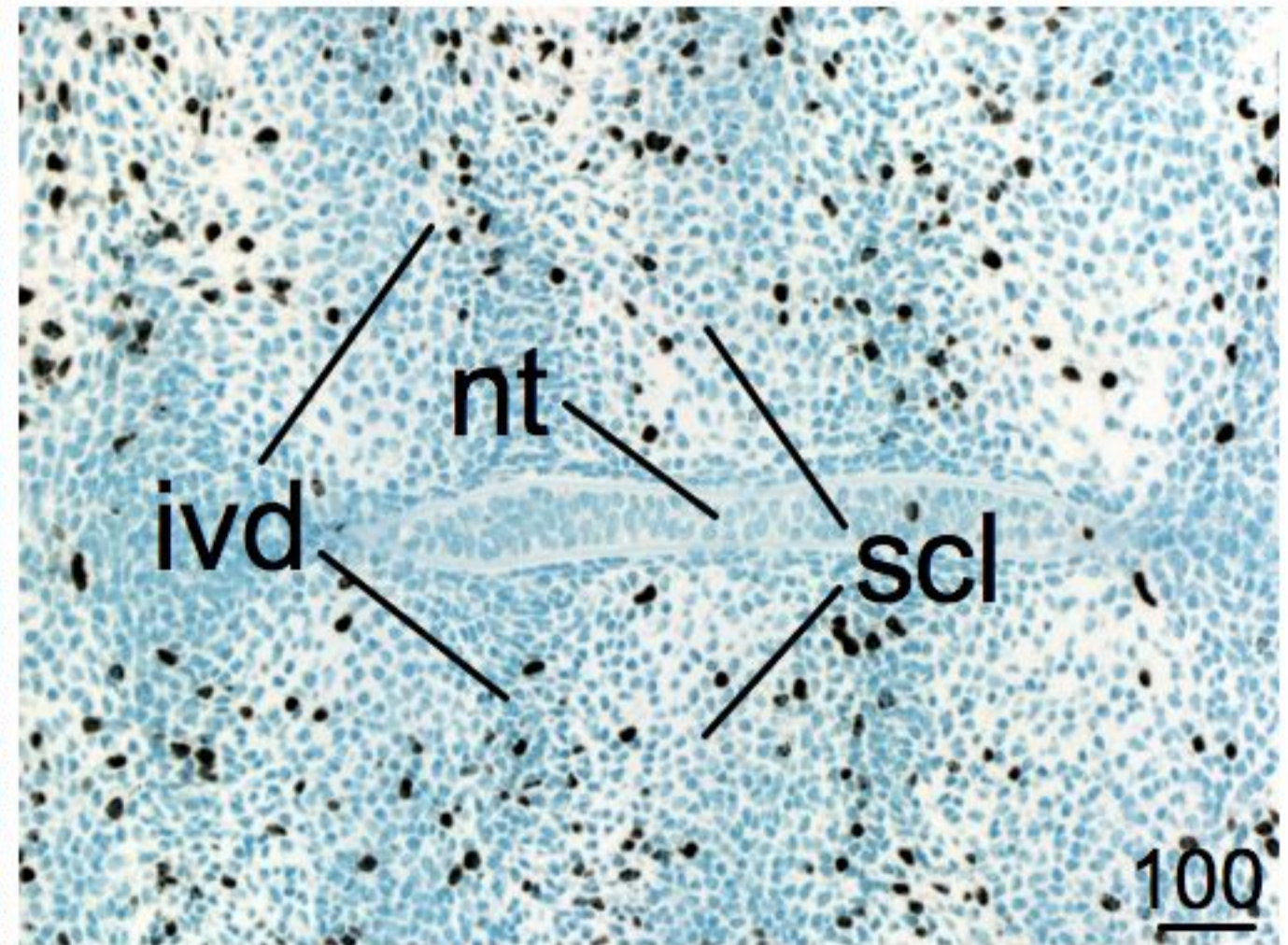

sagittal

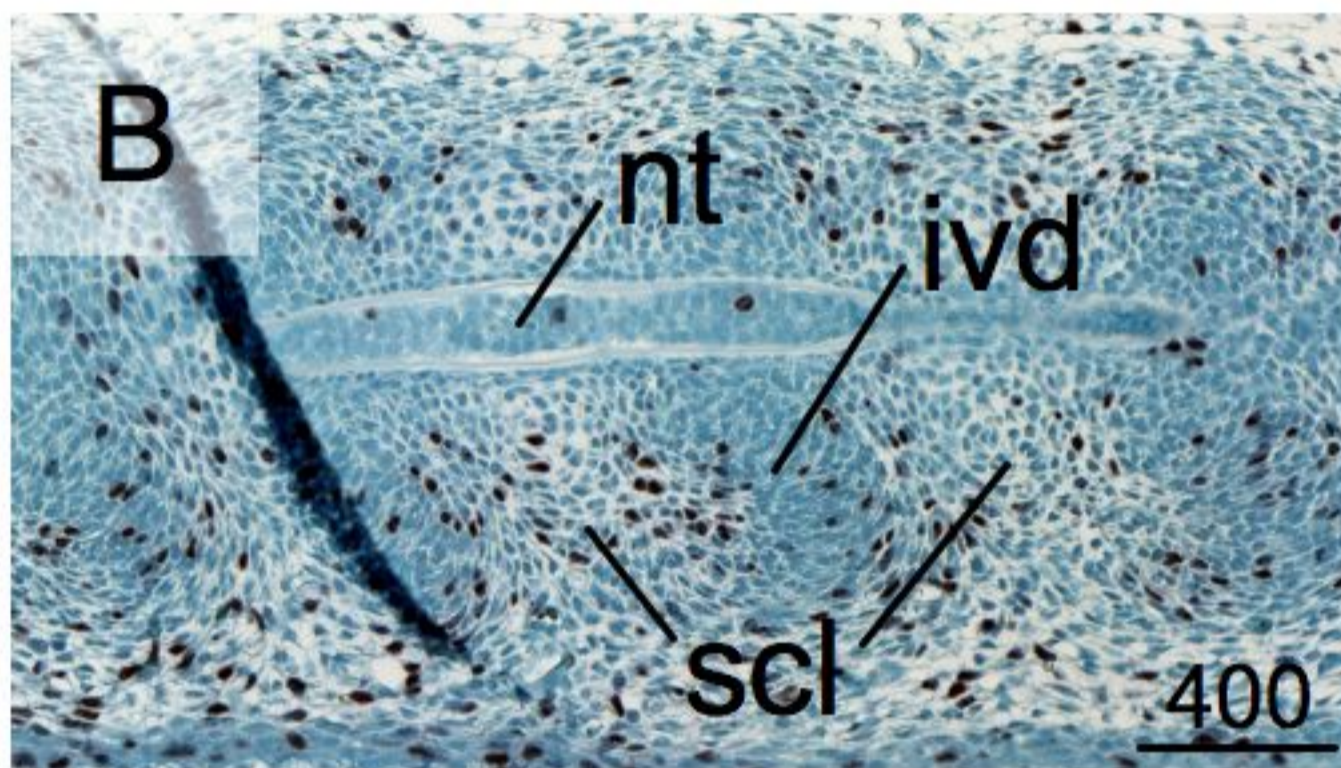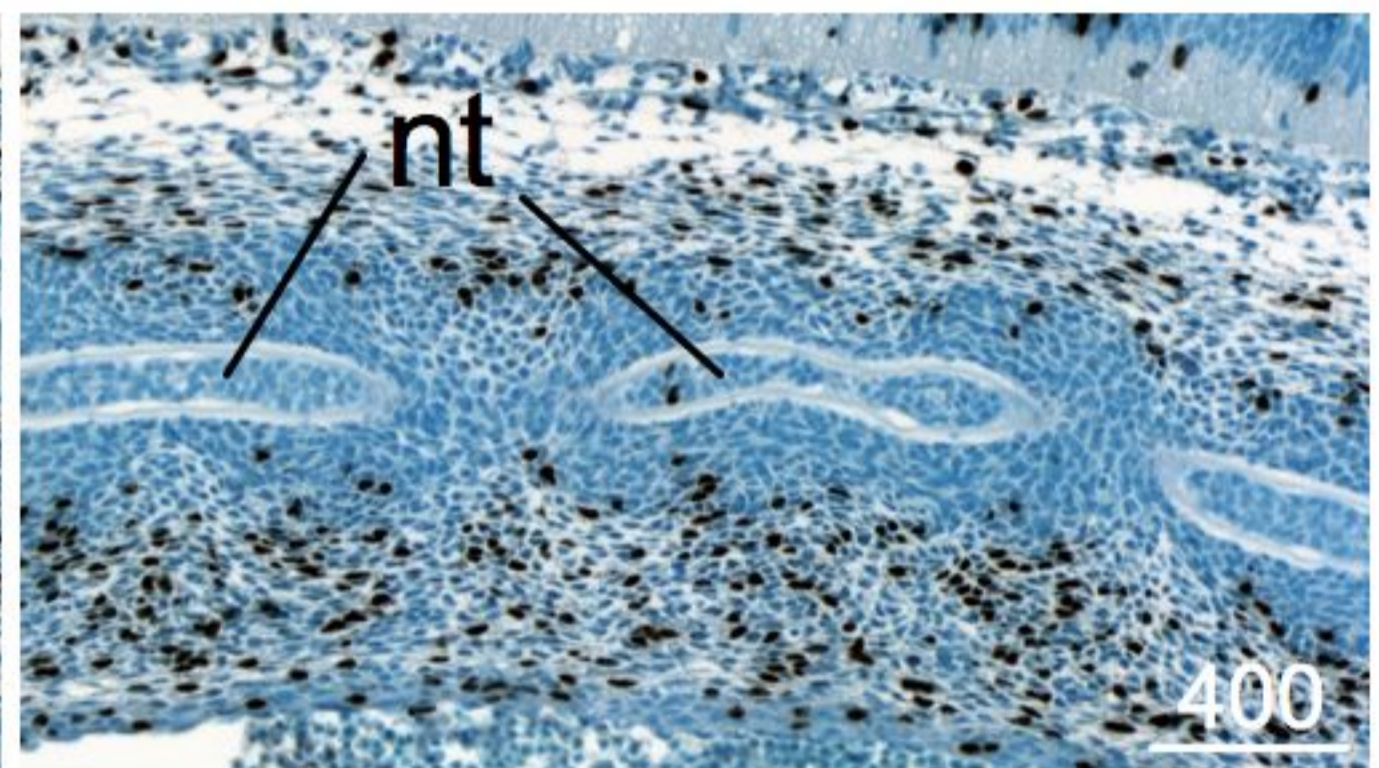

frontal

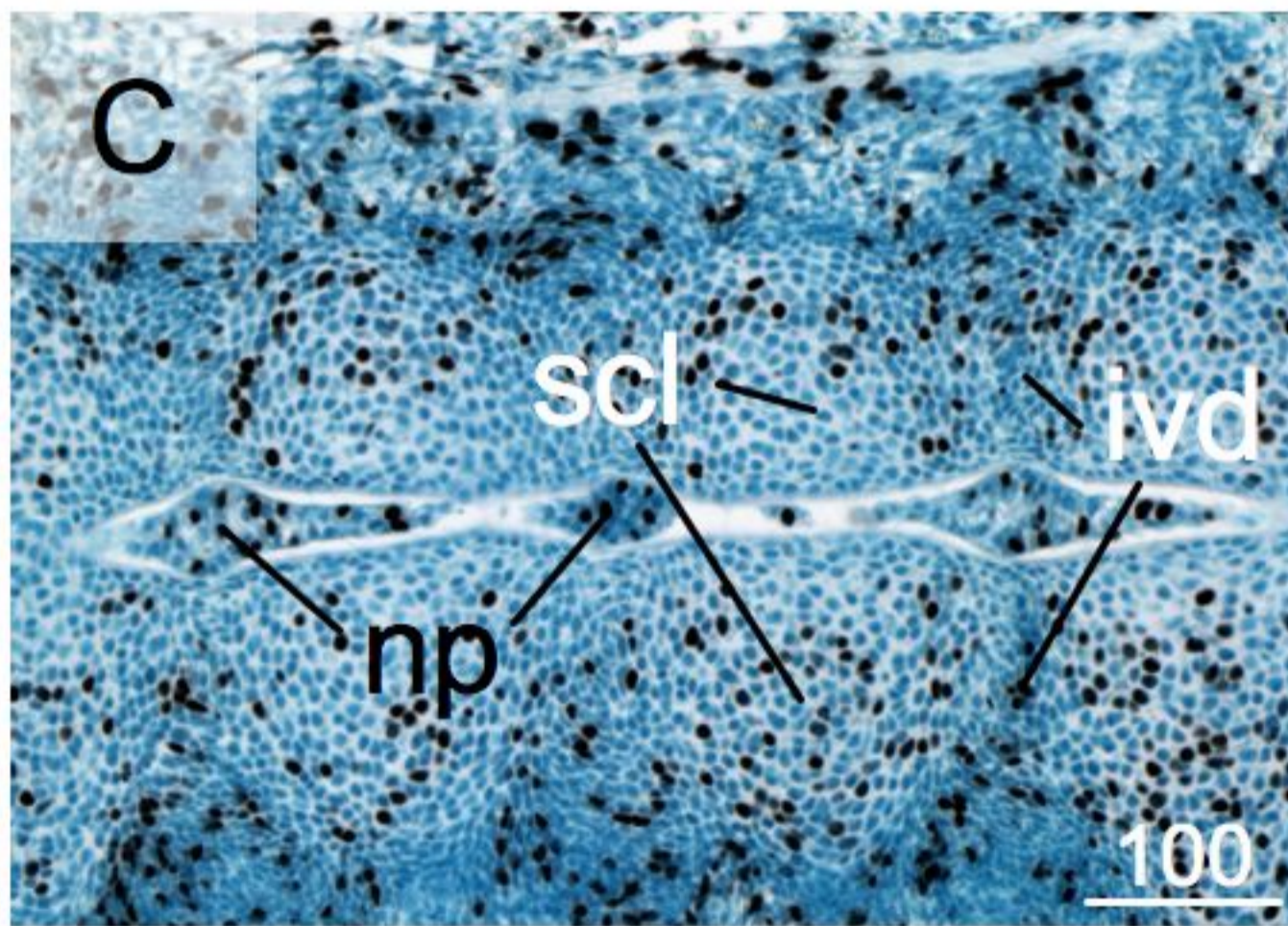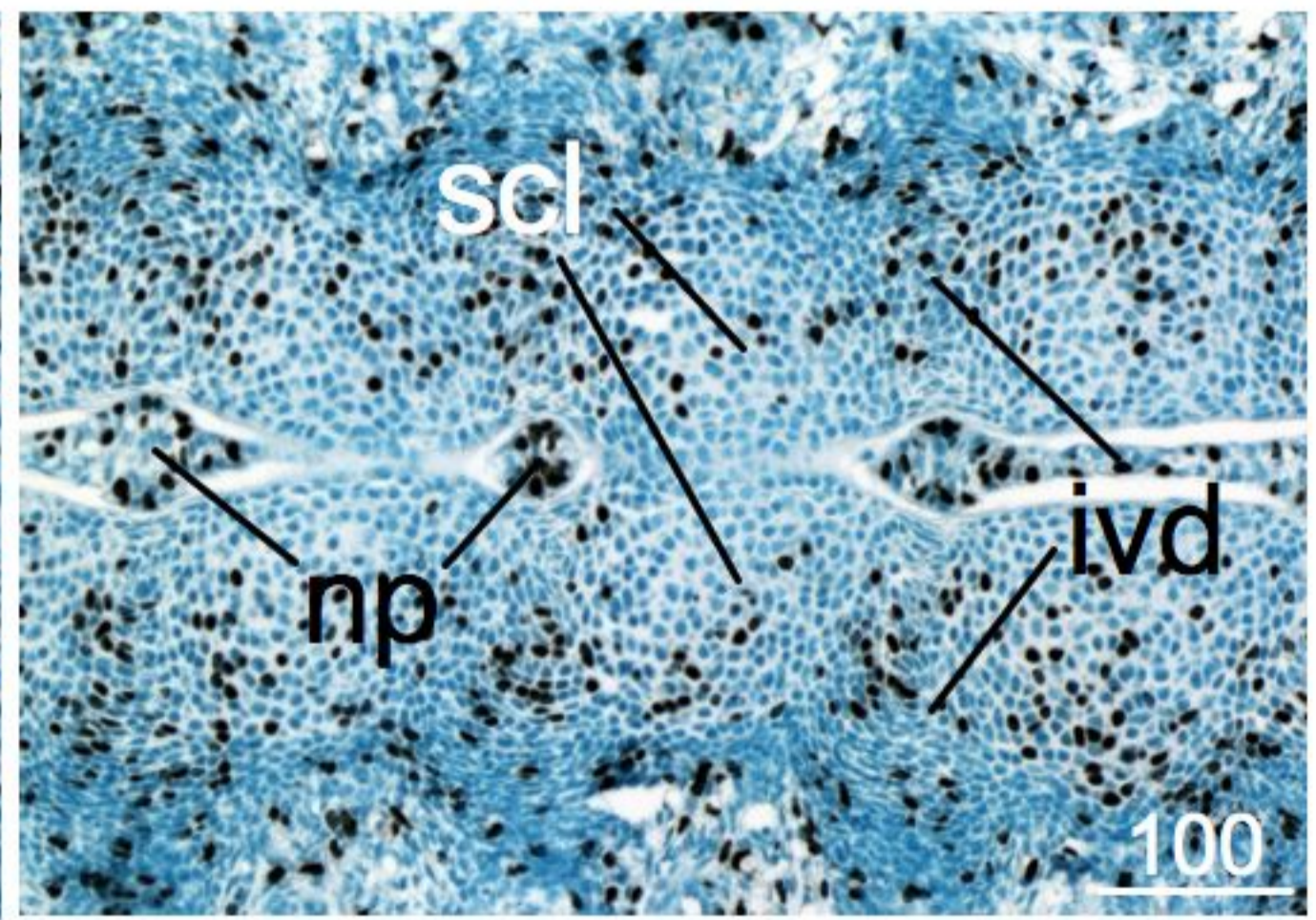

sagittal

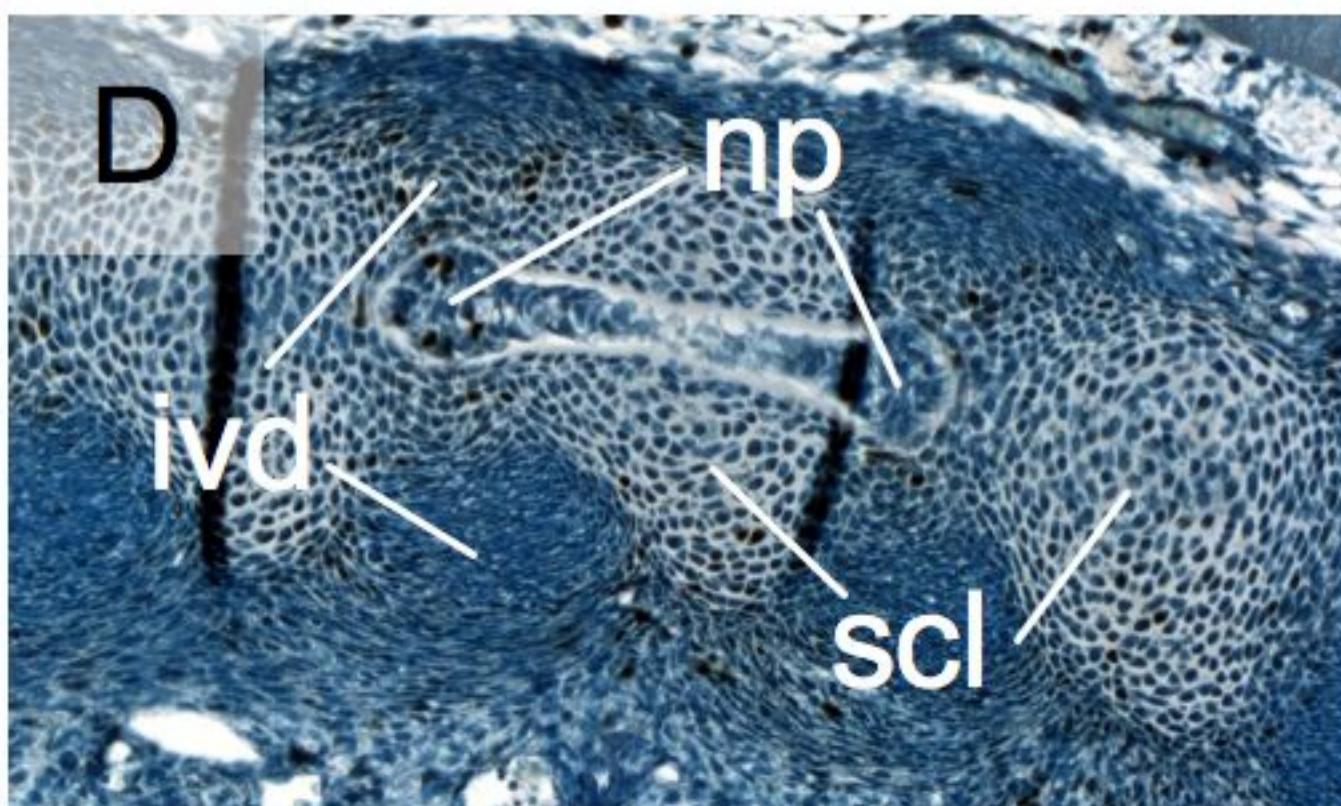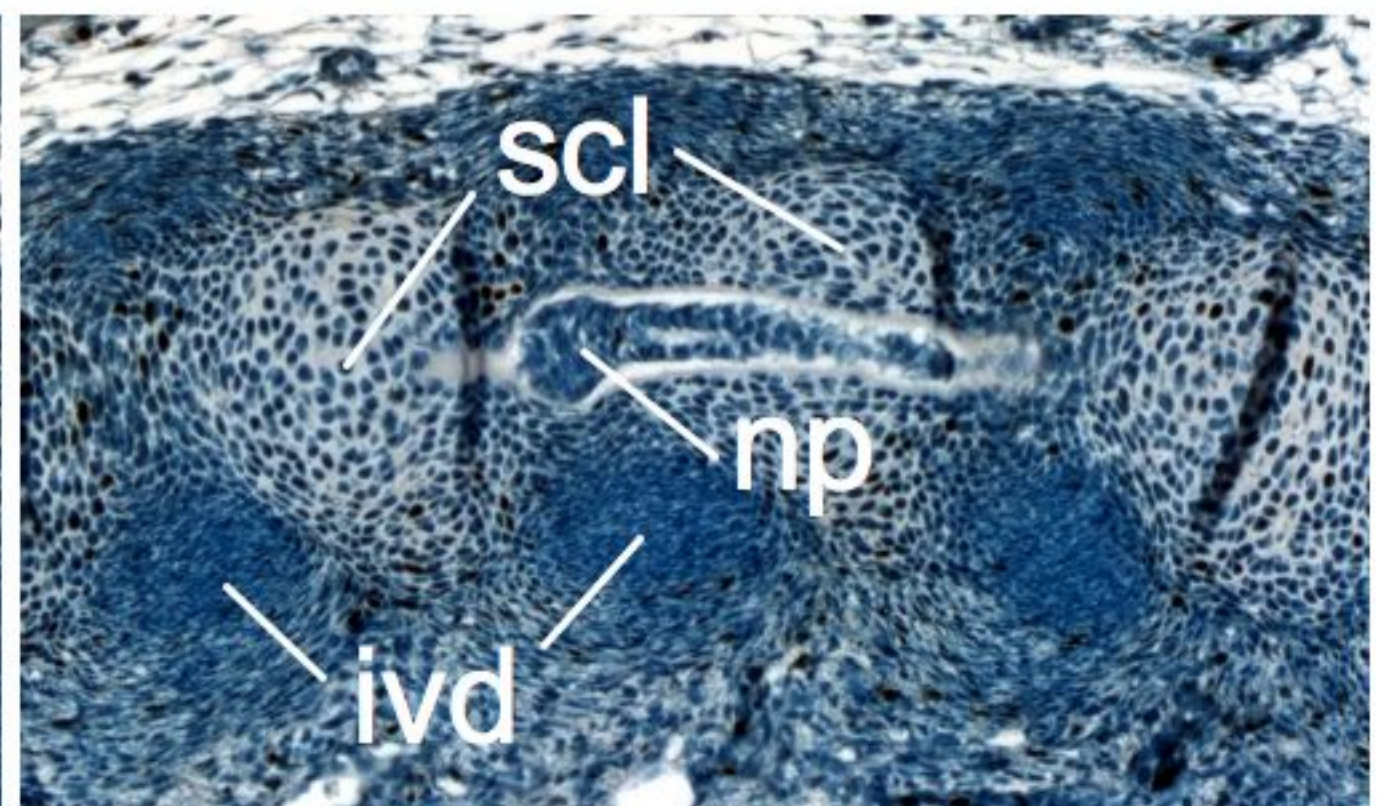

E 12.5

E 13.5

Supplement: Additional File 3 — BrdU assay in frontal (A and C) and mid-sagittal sections (B and D) of E12.5 and E13.5 wild-type (wt, left) and transgenic (tg, right) embryos. (A) shows frontal sections at the level of the notochord (nt) through a wt (left) and transgenic (right) embryo at E12.5. Presumptive regions of intervertebral discs (ivd) and the region of the sclerotome (scl) can be histologically distinguished. There is no difference in the number of BrdU labelled cells if regions of ivd and scl are compared. The sectioned transgenic embryo (right) shown in panel (B) has a severe phenotype: There is no clear metameric sequence of ivd and scl regions. At E13.5 (C and D) the formation of the nucleus pulposus (np) initiates. Also at this stage there is no change in the number of BrdU labelled cells if scl and ivd are compared in wt and tg embryos. Numbers above scale bars indicate the size in μm. [file 1471-213X-7-68-S3.pdf]

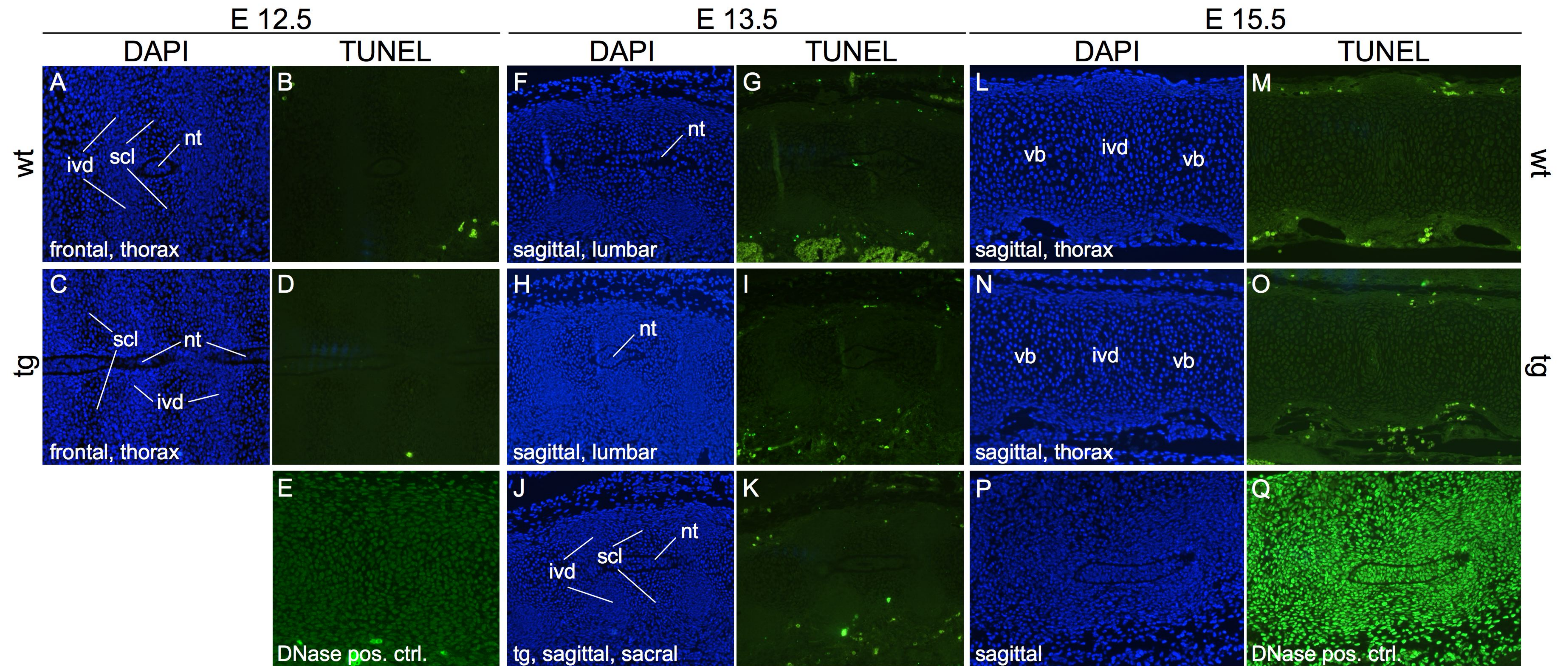

Supplement: Additional File 4 — TUNEL assay and DAPI stainings in sections of E12.5 (A – E), E13.5 (F – K), and E15.5 (L – Q) wild-type (wt) and transgenic (tg) embryos. Panels (A – D) show frontal sections at the level of the notochord in the thoracic region. Notochord (nt), sclerotome (scl), and presumptive intervertebral regions (ivd) are histologically recognizable in DAPI stainings (left panels). Based on TUNEL assays (right panels, B and D) no indication of increased DNA fragmentation was evident in any particular region of the section. Panel (E) shows a positive control that was DNase treated. Sagittal sections through the lumbar (F – I) and sacral (J, K) regions did not reveal increased fluorescence in particular regions in the TUNEL assay in wt (F and G) and transgenic (H – K) embryos at E13.5. Panels (L – O) show sagittal sections through the thoracic regions of a wt (L, M) and a transgenic (N, O) E15.5 embryo. The presumptive vertebral bodies and a future ivd are discernible in the DAPI stainings. No increased fluorescence was detected in these regions in the TUNEL assay. Panel (Q) shows a DNase treated positive control for the TUNEL assay and panel (P) is a DAPI staining of the same section. [file 1471-213X-7-68-S4.pdf]
